# Investigating the biochemical signatures and physiological roles of the FMO family using molecular phylogeny

**DOI:** 10.1016/j.bbadva.2023.100108

**Published:** 2023-11-04

**Authors:** C.R. Nicoll, M.L. Mascotti

**Affiliations:** a Department of Biology and Biotechnology Lazzaro Spallanzani, University of Pavia, Via Ferrata 9, 27100, Pavia, Italy; b Molecular Enzymology, Groningen Biomolecular Sciences and Biotechnology Institute, University of Groningen, Nijenborgh 4, 9747, AG Groningen, The Netherlands; c IMIBIO-SL CONICET, Facultad de Química Bioquímica y Farmacia, Universidad Nacional de San Luis, Ejercito de los Andes 950, D5700HHW, San Luis, Argentina

**Keywords:** Flavin-containing monooxygenases, Phylogeny, Secondary metabolism, Xenobiotic detoxification, Monooxygenases

## Abstract

Group B flavin-dependent monooxygenases are employed in swathes of different physiological functions. Despite their collectively large substrate profile, they all harness a flavin-based C4a-(hydro)peroxy intermediate for function. Within this class are the flavin-*containing* monooxygenases (FMOs), representing an integral component within the secondary metabolism of all living things – xenobiotic detoxification. Their broad substrate profile makes them ideal candidates for detoxifying procedures as they can tackle a range of compounds. Recent studies have illustrated that several FMOs, however, have unique substrate profiles and differing physiological functions that implicate new roles within secondary and primary metabolism. Herein this article, by employing phylogenetic approaches, and inspecting structures of AlphaFold generated models, we have constructed a biochemical blueprint of the FMO family. FMOs are clustered in four distinct groups, with two being predominantly dedicated to xenobiotic detoxification. Furthermore, we observe that differing enzymatic activities are not constricted to a ‘golden’ set of residues but instead an intricate constellation of primary and secondary sphere residues. We believe that this work delineates the core phylogeny of the Group B monooxygenases and will prove useful for classifying newly sequenced genes and provide directions to future biochemical investigations.

## Highlights

• FMOs phylogeny is a powerful predictive source of new enzymes functionality.
• FMOs evolution is dominated by gene duplication and losses across the tree of life.
• Enzyme activity in FMOs is not constricted to a golden set of residues.
• FMOs activity is governed by a network of first and second sphere residues.
• Group b monooxygenases are the tDNBD monooxygenases.

## Introduction

**Scheme 1:**
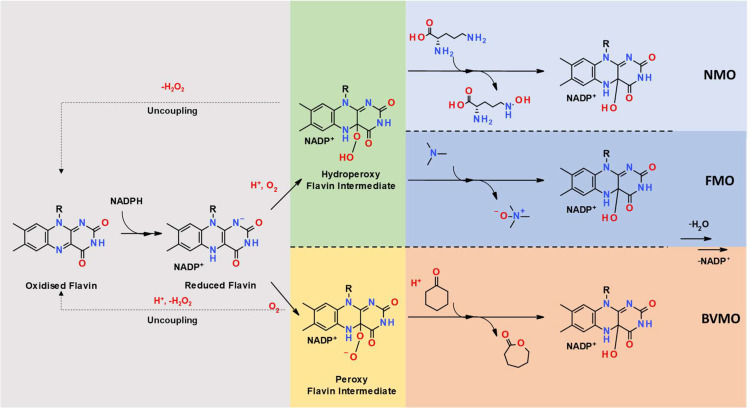
Mode of catalysis for Group B Flavin-dependent monooxygenases – NMOs, FMOs and BVMOs. For simplicity, one substrate is shown for each enzyme. Ornithine, trimethylamine and cyclohexanone are shown for NMOs, FMOs and BVMOs, respectively. The uncoupling reaction representing the unproductive decay of the (hydro)peroxy intermediates and release of hydrogen peroxide is shown as a dashed line.

Group B flavin-dependent monooxygenases are ubiquitous in all forms of life and involved in both primary and secondary metabolisms [1]. Their physiological roles include host-defense, whether toxin production or elimination, and the biosynthesis of natural products such as growth hormones, siderophores and antibiotics. Collectively, Group B monooxygenases (also known as tDNBD -two dinucleotide-binding domain- monooxygenases [2]) can be divided into three enzyme groups: Baeyer-Villiger monooxygenases (BVMOs), N-hydroxylating monooxygenases (NMOs) and flavin-containing monooxygenases (FMOs) [3], also named as subgroups B1, B2 and B3 respectively, according to Paul and co-workers [4]. They are all one-component systems and possess a paired Rossmann-fold that accommodates both flavin adenine dinucleotide, FAD, and reduced nicotinamide adenine dinucleotide phosphate, NADPH [5,6]. Additionally, each of these domains have well conserved sequence motifs: the FAD and NADPH binding domains both possess the motif, (GXGXXG), whilst FMOs also consist of an additional sequence, (FXGXXXHXXXY/F), which represents the linker region connecting the two domains together [7,8]. Despite their disparate tasks, all these enzymes harness a peroxyflavin intermediate to orchestrate chemical transformations [9] (Scheme 1): the NMOs and FMOs employ a protonated hydroperoxyl-flavin intermediate for catalysis, whereas the BVMOs use the deprotonated equivalent peroxy-flavin. The reaction starts with the reduction of FAD by NADPH. As the reduced coenzyme docks into the active site, a complex rapidly forms between NADPH and oxidized FAD followed by the *pro-R*-hydride of NADPH being donated to the N5 position of the FAD creating a reduced FAD-oxidized NADP^+^ system [10]. This complex reacts with oxygen forming the (hydro)peroxy-flavin which is stabilized by the 2′-OH of the bound NADP^+^.

Within FMOs, heteroatom oxygenations are the predominant reaction type. These heteroatoms are typically soft nucleophilic centers including N and S atoms and the corresponding substrates vary in reactivity depending on the number and the electronic nature of the substituents attached. Interestingly, several FMOs have now been documented to confer Baeyer-Villiger (BV) oxidation in line with their BVMO counterparts [11]. Our understanding of the physiological roles undertaken by FMOs is well established for the animal and plant kingdoms [8,[12], [13], [14]]. Bacterial FMOs until now have been studied predominantly for their biocatalytic potential owing to their broad substrate range [15,16,17]. Yeast FMOs have been postulated to play an integral role in controlling the redox potential of the endoplasmic reticulum [18]. Yet, this represents one small portion of all known yeast FMOs.

Across the enzyme family, only a small cluster of FMOs have been structurally resolved and are typically bacterial in origin. However, recently, Nicoll and co-workers were able to resolve the structures of four mammalian FMOs thanks to the enhanced stability introduced by ancestral sequence reconstruction [19]. This result provided new insights into detoxifying mechanisms conducted by membrane binding FMOs. The challenge with profiling the FMO family is underscored by the range of substrate sizes metabolized and the number per enzyme. This is further pronounced by the varying activities in the FMO arsenal. Nevertheless, great strides have being made over the last several decades documenting the various activities conducted by each FMO and even some structures solved [4,20]. In this work we pool together and categorize our current knowledge on this vast enzyme family by performing evolutionary studies and structural analyses. We aim to provide a roadmap for the discovery and characterization of novel FMOs.

## Results

### FMOs are not monophyletic and show a complex taxonomic distribution

**Fig. 1:**
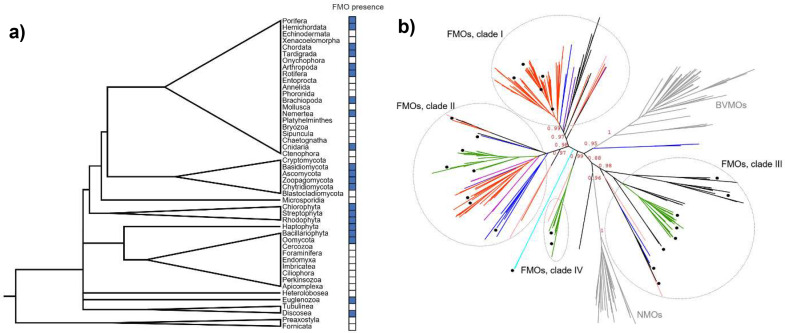
**a)** FMOs distribution in Eukarya domain; **b)** ML tree colored by taxonomy: metazoan (orange), fungi (blue), plants (green), hacrobia (pink), lophotrochozoa (bright pink), euglenozoan (cyan), amoeba (purple), Bacteria and Archaea (black). The black circles at the tips indicate the experimentally characterized members of each clade. The external groups are shown in gray branches. The support at the branches (TBE) is presented for key divergences in the phylogeny.

The evolutionary history of Group B monooxygenases is complex, as it has been greatly influenced by gene duplications and losses [21]. In order to have an accurate and complete picture on the evolution of FMOs specifically, we designed a strategy to build a dataset ensuring representativity and robustness. We vetted from each domain of life all phyla/classes by selecting two representative species with complete genomes in databases to perform homology searches. When no complete genomic information was available, the species with the highest level of sequence coverage were mined, otherwise the whole phyla was mined (Table S1). We observed that among Eukarya, FMOs are well distributed, although there are some phyla missing them, mainly from the SAR supergroup as well as from the metamonada and metazoan groups (Fig. 1a, Fig. S1). When analyzing prokaryotic domains, a more restricted distribution is observed. FMOs are found in cyanobacteria, actinobacteria, proteobacteria, spirochaetes, rhodothermaeota and bacteroidetes from Bacteria and only in Halobacteria class from Archaea domain.

The maximum likelihood (ML) inferred phylogeny showed a topology in agreement with the one reported in 2015 [21]. FMOs appear as paraphyletic whereas BVMOs and NMOs are monophyletic groups splitting from FMO-like ancestors. Four well-supported FMO clades were identified. The taxonomic distribution observed shows a patchy pattern (Fig. 1b, Fig. S2), expected for gene duplication and loss as the main mechanism of FMOs evolution, and not excluding the possibility of some horizontal gene transfer events.

Clade I (TBE 0.97) is composed largely by metazoan as well as some fungal, bacterial and oomycetes sequences. No plant sequences are found among this group. Interestingly, a large part of the metazoan FMOs included here, from vertebrates and insects, form a well-supported clade (TBE 0.99) and its topology matches the species tree. The other species sequences, although undoubtedly belonging to this clade (TBE 0.97 at the deep branching point), they are placed in subgroups with lower support values, which suggests that a different topological arrangement could possible.

Clade II (TBE 0.97) is the sister clade of Clade I and is composed by plant, fungi, oomycetes and metazoans from the tardigrada, brachiopoda, nematoda and nematostella groups. This clade contains most of the biochemically and structurally characterized FMOs to date (Table 1, Table S2). For example, the insect FMOs involved in the detoxification of pyrrolizidine alkaloids from plants, PNO from *Zonocerus variegatus* [22] and SNO from *Tyria jacobaeae* [14], the yeast FMOs from *S. cerevisiae* [23] and *S. pombe* [24] devoted to redox regulation, the garlic FMO [25] involved in secondary metabolites biosynthesis and the bacterial FMOs whose physiological role is unknown.

**Table 1:** Representative FMOs from each clade experimentally characterized to date.

Phylogenetic clustering	Enzyme name	Accession code*	PDB code(s)	Organism	Reference(s)
Clade I	AncFMO1	UZZ64694.1	7al4	Synthetic construct (mammalian ancestor)	[30]
	AncFMO2	UZZ64691.1	6sem	Synthetic construct (mammalian ancestor)	[19]
	AncFMO3–6	UZZ64692.1	6se3	Synthetic construct (mammalian ancestor)	
	AncFMO5	UZZ64695.1	6sek	Synthetic construct (mammalian ancestor)	
Clade II	mFMO	Q83XK4	2vqb, 2vq7	*Methylophaga aminisulfidivorans*	[31]
			2xlr		[32]
			2xve		[33]
	ZvPNO (PNO)	L0N8S9	5nmw	*Zonocerus variegatus*	[22]
	SNO	Q8MP06	–	*Tyria jacobae*	[14]
	FMO-GSOX1	OAP12743	–	*Arabidopsis thaliana*	[34]
	yFMO (FMO1p)	AJU16467	–	*Saccharomyces cerevisiae*	[23]
	SpFMO	Q9HFE4	1vqw	*Schizosaccharomyces pombe*	[35]
			2gv8		
	AsFMO	BAS32646	–	*Allium sativum*	[25]
	RnTmm	A3SLM3	5gsn	*Roseovarius nubinhhibens*	[36]
	NiFMO	A0A063Y6V3	6hns	*Nitrincola lacisaponensis*	[16]
	Tmm	B6BQB2	7d4k	*Candidatus Pelagibacter sp*. HTCC7211	[37]
Clade III	FMOF	Q0S8V1	–	*Rhodococcus jostii*	[26]
	FMOA	A0A1H4IID2	–		
	FMOB	Q0SBE9	–		
	YUCCA 1 (OsFMO)	XP_015631023	–	*Oryza nivara*	[38]
	YUCCA1	OAP00458	–	*Arabidopsis thaliana*	[39]
Clade IV	AtFMO1	OAP18020	–	*Arabidopsis thaliana*	[27]
	FOS1	QNT35807	–	*Phlebodium aureum*	[28]
	FOS1	QNT35806	–	*Pteridium aquilinum*	
–	TcFMO	XP_817,059	–	*Trypanosoma cruzi*	[29]

⁎ Genbank or Uniprot accession codes are given.

Clade III (TBE 0.91) is the closest to NMOs (TBE 0.88). This is the most divergent group, and it is composed almost exclusively by the YUCCAs from plants and some prokaryotic FMOs. The bacterial FMOs found here, were proposed to form a different class already in 2013 due to their different biochemical properties and divergent clustering [26].

Finally, Clade IV (TBE 0.99) is the least diverse and populated. It is exclusively formed by plant sequences and just a single sequence from the hacrobia *Emiliania huxleyii*. Members of this clade include the FMO1 from *Oryza sativa* and its ortholog from *A. thaliana*, described to be involved in plants systemic immunity [27] and the recently characterized fern FOS1 involved in secondary metabolites biosynthesis [28].

Interestingly the FMO from *T. cruzi*, characterized in 1987 [29], is found clustered with a *Leishmania* sequence, next to Clades II and IV, but not belonging to them. When constructing the tree with preliminary datasets we observed that these branches changed their location in the phylogeny. Hence, we cannot confirm to which group they belong. Nevertheless, according to the experimental data TcFMO behaves as a canonical FMO (Table 1, Table S2).

Finally, close to the BVMOs clade, there are a few fungal sequences early diverging. These sequences do not harbor the typical BVMO sequence motifs [26] and do not display the archetypal BVMO structural features so as to be included in their clade. However, they also include mutations in the canonical FMO fingerprint.

### FMOs have a common structural fold

**Fig. 2:**
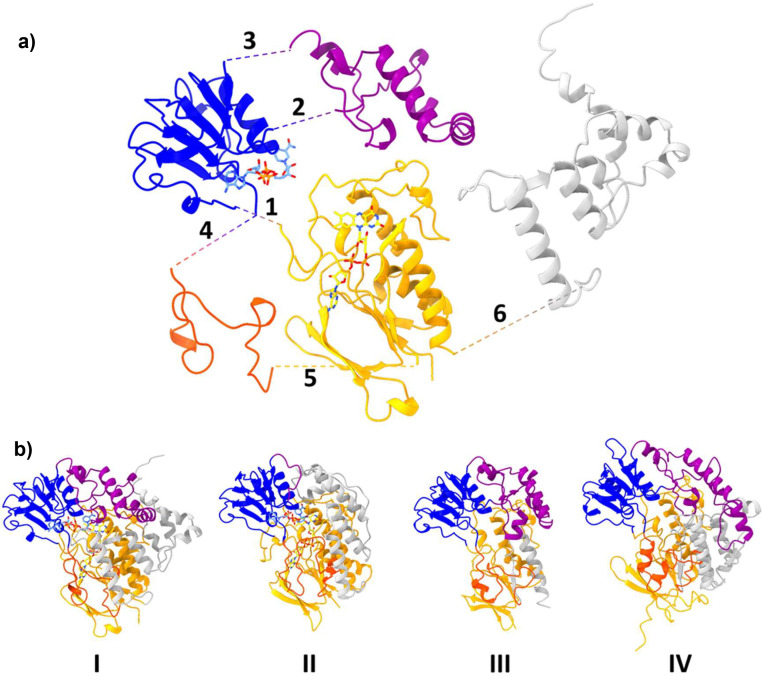
**Structural topology of the FMO family. a)** Dissection of the FMO structure into its individual domains. The highly conserved FAD- and NADP-binding domains that collectively illustrate the paired-Rossmann fold are shown in orange and blue, respectively. The NADP-binding domain insertion, the flexible loop domain and the C-terminal domain are shown in purple, dark orange and dark gray, respectively. The mammalian FMO5 crystal structure is used as a model. Domains connect as shown by the numbered branches in order of amino acid sequence. **b)** Structural topology for each FMO clade is shown by four different clade representatives: mammalian AncFMO5 (PDB: 6sek, Clade I), *Methylophaga aminisulfidivorans* mFMO (PDB: 2vq7, Clade II), *Rhodococcus jostii* FMOA (WP_011593985, Alphafold2, Clade III) and *Arabidopsis thaliana* FMO1 (OAP18020, Alphafold2, Clade IV), respectively. Cofactors FAD and NADP^+^ are shown in yellow and cornflower blue, respectively.

FMOs possess a highly conserved paired-Rossmann fold that accommodates the adenosine-based molecules FAD and NADPH (Fig. 2). Bridging these two protein domains are three additional motifs that vary across the FMO family tree considerably in size, porosity, and charge and collectively build the various tunnels, cavities, and chambers to channel and facilitate substrate turnover (Fig. 2, Fig. S4). These three sections include: i) an NADPH-binding domain insertion, ii) a flexible loop domain and iii) the C-terminus. The flexible loop domain does not vary greatly in length across the FMO family but adopts several different conformations. Alternatively, the other two domains are the most divergent and change significantly across every clade. This domain construction is also present in the closely related BVMO and NMO families (Fig. S4). BVMOs possess a longer NADPH-binding insertion, reaching approximately 120 amino acids, compared to 80–100 residues as seen for clades I, III, IV and the NMOs. NMOs, however, do not possess the C-terminal domain. Instead, the NADPH-binding insertion stretches downward, across the protein surface, presenting a large substrate groove for active site access. Clade III FMOs share a monophyletic ancestor with the NMOs and similarly do not possess a C-terminal domain.

In clade I, the NADPH-binding domain insertion provides membrane binding capacity and tunnels for substrate transit, whilst clade II FMOs possess a small loop with no clear role. Indeed, this shortened NADPH insertion accounts for the difference in polypeptide length between clade I (*ca*. 500 residues) and clade II (*ca.* 450 residues) FMOs. Clade III FMOs are the shortest of the family (420 residues, excluding FMOF discussed below), whilst clade IV FMOs are more similar to clade I FMOs (*ca.* 520 residues).

Clade III is the most divergent of the four and includes some members harboring an additional domain to the common core. These enzymes (*e.g*.: FMOF from *Rhodoccocus jostii*) show a N-terminal extension corresponding to the domain covering the whole length of the nuclear transport factor 2 (NTF2). NTF2 protein is a nuclear envelope protein facilitating protein transport into the nucleus (CATH Superfamily 3.10.450.50). This additional domain seems not to have an influence on enzyme catalytic properties [26].

Mammalian FMOs present in clade I were observed to possess a highly hydrophobic surface, that stretches across the C-terminal domain and an NADP-binding domain insertion, that provides accessible channels for hydrophobic substrates residing in the membranes. These members were observed to be membrane-bound and are equipped with an extended C-terminal helix that drills into the membrane and latches the enzyme firmly onto the phospholipid bilayer [19]. With the addition of AlphaFold2, inspecting the predicted surface charge of other members of clade I reveals that they too possess a highly hydrophobic C-terminal helix and a large hydrophobic surface and are very likely membrane residing. Inspecting the surface charges of the other FMO clades, we observe that clade IV, similarly to clade I, seems to possess hydrophobic protruding features close to substrate entry points. These FMOs could be predicted to partially interact with the membrane, however, Thodberg *et al.*, highlighted that PaqFOS1 did not require detergent for activity and it eluted as a homodimer [28]. This would suggest that the hydrophobic surface represents a potential dimerization interface. On inspection, clades II and III do not appear to be membrane dwelling and this is well supported by literature.

### FMO tunneling and active site features

**Fig. 3:**
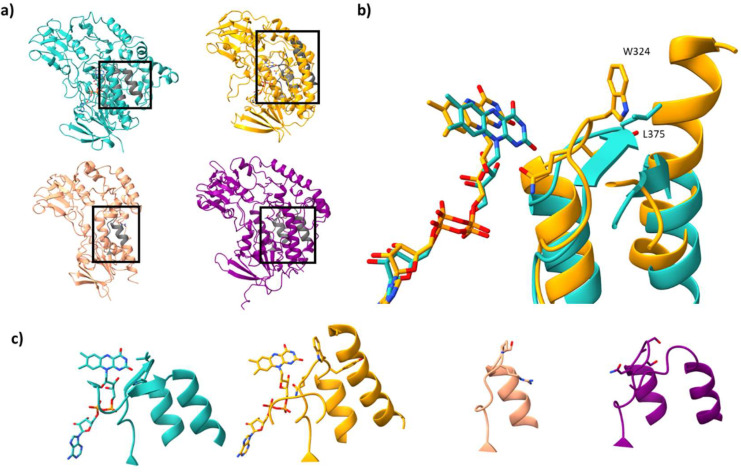
**Substrate gatekeeper features across the FMO phylogeny. a)** Substrate entry points for each FMO type are shown. Secondary structures shown in gray correspond to the gatekeeper motif permitting substrate/product entry/exit. **b)** superposition of the class I and II FMO crystal structures emphasizing the steric blockade introduced into the latter that restricts molecular transit. The gatekeeper residue in FMO5, L375, is shown, alongside the clade II blockade residue, W324, that stacks against the elongated alpha helix. **c)** Close up view of the gatekeeper motifs for each FMO class. Gatekeeper residues present on the gatekeeer loop are shown. Mammalian AncFMO5 (PDB: 6sek, Clade I), *Methylophaga aminisulfidivorans* mFMO (PDB: 2vq7, Clade II), *Rhodococcus jostii* FMOA (WP_011593985, Alphafold2, Clade III) and *Arabidopsis thaliana* FMO1 (OAP18020, Alphafol2, Clade IV), are colored in light sea green, orange, light salmon and purple, respectively.

**Fig. 4:**
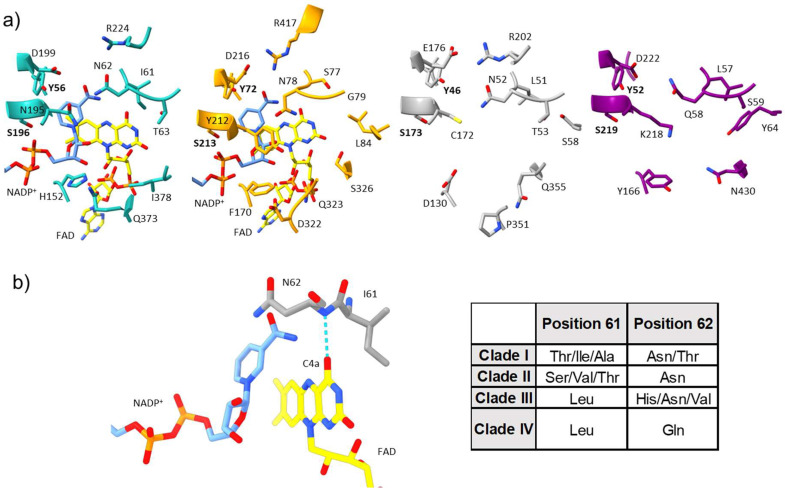
**Comparison of the active sites across the FMO phylogeny. a)** Active sites of representative FMOs for each clade. Active sites are shown for the mammalian AncFMO5 (PDB: 6sek, Clade I, light sea green), *Methylophaga aminisulfidivorans* mFMO (PDB: 2vq7, Clade II, orange), *Rhodococcus jostii* FMOA (WP_011593985, Alphafold2, Clade III, light gray) and *Arabidopsis thaliana* FMO1 (OAP18020, Alphafold2, Clade IV, purple), from left to right, respectively. Residues that are conserved throughout the FMO phylogeny are shown in bold. **b)** Active site of mammalian AncFMO5 is shown with the two key residues (dyad) Ile61 and Asn62 shown. The hydrogen bond between the isoalloxazine and the peptide backbone is shown in cyan. The C4a position is labelled. Cofactors NADP^+^ and FAD are shown in cornflower blue and yellow, respectively. Table documenting the various residues found across the FMO phylogeny at each dyad position.

Considering the vast number of substrates transformed by the FMO family, we set out to investigate whether they possess key differences among their tunnel and active site architectures (Fig. 3. and Fig. 4). We first used the crystal structures of the mammalian class I FMOs as a reference. Elucidation of these structures showcased several different tunnels with many different entry and exit points on the protein surface [19,30]. One structure in particular, AncFMO3–6, displayed a gatekeeper residue, Leu375, that is found at the apex of a small loop. After surpassing this loop, several different tunnels emerged across each FMO. Inspecting structures from clades II, III and IV showed similar features with the same loop present, originating from the C-terminal domain, in all cases. Despite not possessing the same gatekeeper residue, we postulate that this loop mediates substrate/product entry/exit. Indeed, each clade presented similar entry points for substrates (Fig. 3a), fabricated by both the NADP-binding insertion and C-terminal domains. This feature is also observed for BVMOs (Fig. S5). Unlike clades I and IV, clade III has a very short C-terminal domain that, as a result, exposes this loop to the surrounding solvent and significantly truncates the tunnel (Fig. 3a). Of note, NMOs do not show similar structural features in this regard. The stretched NADPH-binding domain in NMOs creates a cavity that renders the isoalloxazine readily accessible (Fig. S5). Clade II FMOs are more like NMOs in terms of substrate uptake. Firstly, the isoalloxazine ring is exposed to the exterior solvent and not shielded by the protein scaffold on account of its shortened NADPH-binding domain. Secondly, whilst still possessing the same gatekeeper loop found in the other FMOs, it is instead nestled against a large helix, present in the C-terminal domain, that stretches across and blocks the formation of a potential tunnel (Fig. 3b). This finding suggests that clade II FMOs have evolved an entirely different mechanism for substrate uptake that passes through a funnel-like active site cavity.

Next, we turned to the FMO active sites to evaluate whether any clades possess a unique core. Inspecting several FMO active sites revealed that only two residues, Ser196 and Tyr56 (mammalian AncFMO5 numbering), that lie on the edge of the active site close to the nicotinamide ring of the coenzyme, are conserved (Fig. 4a). Moreover, several residues expected to coordinate with the amide functional group attached to the nicotinamide ring of NADPH show a high degree of chemical conservation including Asp199, that in clade III FMOs is a glutamate residue, and Arg224 (that is however absent in clade IV FMOs). Interestingly, across the FMO clades, the amino acid at position 195, that has been coined as the back door residue to the nicotinamide ring, shows little conservation; clades I, II, III and IV FMOs possess either asparagine, tyrosine, cysteine, or lysine, respectively (Fig. 4a). The aforementioned residues seem to be important for coenzyme binding and may play critical roles in substrate binding. For example, both Tyr212 (clade II) and Lys218 (clade IV) can act as hydrogen bond donors and extend into the substrate binding site. Alternatively, Asn195, is within hydrogen bonding distance to the 2′-OH ribose of NADPH and may be important for intermediate stability. Similar characteristics are observed for the BVMO and NMO clades (Fig. S6).

A two-residue dyad, located above the isoalloxazine ring at positions 61 and 62, however, has been shown to be important for activity (Fig. 4b). The latter is predominantly asparagine across clades I-III with the presence of threonine, histidine and valine appearing in sparse sequences. Clade IV FMOs show high conservation of a longer, yet chemically equivalent, glutamine; similarly, NMOs possess a glutamine in this position (Fig. S6). Both asparagine and glutamine are postulated to interact and stabilize the C4a-intermediate and mutating this residue in mFMO resulted in abolished activity [6]. Considering that both clade I and III FMOs are able to perform both N/S and BV oxidations, whilst essential for building the intermediate, this residue is not solely responsible for the final protonation state obtained that gives rise to either function. BVMOs alternatively possess an aspartate residue in this position that forms a hydrogen bond with an intruding Arginine residue that is speculated to stabilize both the negatively charged peroxy-flavin and Criegee- intermediates (Fig. S6).

Site 61 on the other hand, shows strict conservation for clades III and IV with a Leu, while clades I and II present many residues including threonine, alanine, serine, valine and isoleucine (Fig. 4b). Of interest, all BV performing enzymes possess either a leucine or isoleucine at this site. Indeed, Bailleul and Yang showed that this residue was imperative for introducing BV activity in ancestral clade I FMOs [40].

YUCCA1 from *Oryza nivara* (clade III FMO), possesses a highly similar active site to the mammalian AncFMO5 crystal structure (clade I FMO) and can perform BV oxidations. On inspection, the major difference near the expected flavin-peroxy intermediate is the histidine residue (His64) hanging over the C4a center of the isoalloxazine ring in place of a highly conserved asparagine residue. The work by Bailleul and Yang illustrated that Baeyer-Villiger monooxygenase activity can be introduced through an epistatic effect between three residues: a hydrophobic isoleucine located on the *si*-face of the isoalloxazine ring (*re*-face posing toward the oncoming substrate), and two histidine residues lining the NADP^+^ binding pocket [40]. In the YUCCA structure, we see a leucine residue occupying the same space as the respective isoleucine residue, but the other domains present an entirely different structural topology with respect to mammalian AncFMO5. Of note, YUCCA1 from *Arabidopsis thaliana* possesses an almost identical active site with respect to its orthologue found in *Oryza nivara*, however, instead performs N/S oxygenations, suggesting that the overhanging histidine residue is not enough to confer BV activity. This finding corroborates the importance of secondary sphere residues in enzyme function.

### BVMOs and NMOs: the derived groups of flavin containing monooxygenases

The sister clades, BVMOs and NMOs, can be considered as functionally specialized clades of FMOs. BVMOs, show a more restricted taxonomic distribution in Eukarya than FMOs, being missing from chordates (except in the tunicate *Oikopleura dioica*) as well as from all higher plants. They display high copy numbers in fungi and in bacteria species. On the other hand, NMOs, show an even scarcer overall distribution and are mostly found in fungi and some bacteria. While BVMOs principally perform BV oxidation as their main function in addition to secondary activity concerning the oxidation of heteroatom containing molecules, the NMOs perform almost exclusively the hydroxylation of non-natural amino acids. This divergent chemistry among both kinds of enzymes, suggests that these groups likely originated from an FMO-like ancestor *via* gene duplication events and further diversified by means of neofunctionalization followed by optimization (this would be the case of BVMOs) or a switch in the substrate specificity (in the case of NMOs). If the taxonomic distribution is taken into account, it seems plausible that the emergence and expansion of BVMOs might be linked to a species-specific niche, like soil ecosystems, and driven by the necessity of detoxifying carbonyl compounds. For NMOs their role seems exclusively linked to the synthesis of siderophores and therefore its diversification must have been in response to that functional niche to be filled.

### FMOs capture the functional diversity of Class B monooxygenases

To date, the physiological roles of known FMOs span detoxification metabolism, secondary metabolite biosynthesis and primary metabolism. Literature has so far implicated members of clades I and II to be predominantly involved in xenobiotic detoxification. However, these FMOs have also been suggested to play additional endogenous roles in metabolism and improving longevity. For example, inactive forms of FMO3 in humans have been found to cause trimethylaminuria, or more commonly known as fish odor syndrome, a disease that results in excessive accumulation of trimethylamine that possesses a rotten fish smell [41]. The importance of FMO3 has been further attributed to its role in trimethylamine N-oxide production, a small osmolyte that has been suggested to reduce the risk of atherosclerosis [42], [43], [44]. Alternatively, an FMO found in *C. elegans*, fmo-2 (a clade I FMO), was demonstrated to improve longevity. Recent work suggests that the FMO reduces methylation events during one carbon metabolism and decreases tryptophan levels. The authors postulate that FMOs may in general serve key roles in diet and demonstrate that these enzymes are not solely involved in xenobiotic metabolism [12,45]. Their importance for metabolism more generally is reflected by their presence in all taxonomic groups.

Alternatively, metazoan species do not possess any FMOs in clades III and IV which are currently not predicted to be involved in detoxification metabolism. Several physiological roles for clade III FMOs remain enigmatic with only the plant YUCCAs being demonstrated to be essential for auxin biosynthesis [39]. There are still some elements of conserved substrate turnover, such as thioanisole, but the extent of conversion is very low, and the ‘true’ substrate may not have been found yet. Indeed, clade III FMOs have a distinct substrate profile (Table 2). Clade IV FMOs follow a similar trend regarding their substrate profile and are the most recently discovered group of FMOs.

**Table 2:** Representative substrate scope of FMOs from each clade.

Cluster	Example	Source (taxonomy)	Substrate	Type of oxidation	Reference(s)
Clade I	FMO1, FMO2, FMO3	mammals		S oxidation	[51]

		N oxidation

FMO5	mammals		BV oxidation	[11]

Clade II	GSOX1	plants		S oxidation	[34,25]

	ZvPNO, SNO	arthropods		N oxidation	[14,22]

bFMO, NiFMO	Bacteria		S oxidation	[15,16]

Clade III	FMOF, FMOA, FMOB	Bacteria		BV oxidation	[52]

		S oxidation (<15%)

YUCCA 1 (OsFMO)	plants		BV oxidation	[38]

	N oxidation	[39]

Clade IV	AtFMO1, FOS 1	plants		N oxidation	[27,28]

–	TcFMO	euglenozoa		N oxidation	[29]

Overall, the main functionality comes from the formation of the C4a-hydroperoxyflavin oxygenating intermediate that allows the electrophilic oxidation of sulfides and amines [46], [47], [48]. Across the phylogeny we see several substrates popping up in different clades such as thioanisole (clades I and III) and trimethylamine (clades I and II) (Table 2). The corresponding products are sulfoxides, disulfides (*via* the formation of the sulfenic acids) and N-oxides. Although, N-hydroxylated products are less frequently observed for FMOs [49] it has also been reported that they can produce oximes [50]. Meaning that they are capable of N-hydroxylation, but a second hydroxylation reaction quickly follows before the final hydrolysis step (Table 2).

## Discussion

In this work we have performed an integrated analysis of the FMO enzyme family from an evolutionary and structural point of view and we have created a biochemical roadmap of the family. For a long time, the enzyme classes among the Group B flavoprotein monooxygenases have been interpreted on the basis of their functional diversity. We now have a clear picture showing that functionality is more overlapped and redundant than expected. Clade I and II harbor the most taxonomic diversity. All eukaryotes that have FMOs seem to be equipped with at least one paralog belonging to these clades, indicating the shared role in detoxification or defense mechanism. On the contrary, Clades III and IV are the least diverse. Paralogs here seem to be involved in more specialized functions, as natural products biosynthesis (at least based on the few members characterized) suggesting that these genes have been under the influence of specific selective pressures, hence likely by a shared ecological niche among the hosts. Therefore, it is cumbersome to disentangle the physiological roles of these enzymes from their paths in evolution. This is beautifully depicted when analyzing plant FMOs for example [53]. Among them, three types of FMOs are found, one devoted to primary metabolite synthesis (*e.g.:* YUCCAs from clade III), and the other two devoted to the production of diverse secondary metabolites (*e.g.:* fern FOS1 from clade IV and garlic AsFMO1 from clade II). Unlike this, some metazoan species, such as insects and nematodes have only two kinds, one devoted to secondary metabolism biosynthesis (*e.g.:* cinnabar moth SNO from clade II) and the other to detoxification metabolism (*e.g.:* fmo-2 from *C. elegans* from clade I).

From a structural perspective, some clear events significantly impacting the core structure of FMOs can be identified based on the phylogeny. All FMOs from eumetazoans in clade I, exclusively including chordata phyla, are predicted to be membrane bound. We posit that as FMOs, though debated to belong to the core proteome, became essential for survival by the end of the Devonian period, a membrane associated copy was preferably retained and this further duplicated into at least 5 functional paralogs (Amphibia can have even 7 paralogs). This membrane-binding feature stems from both the NADP-binding and the C-terminal domains that vary the most across the phylogeny. Indeed, for Clade III FMOs and NMOs, the C-terminal domain was abolished entirely. Using just the NADP-insertion, the NMOs have constructed a substrate cleft that resultantly has high substrate specificity towards amino acids. Alternatively, Clade II FMOs possess an extensively truncated NADP-binding domain insertion and, yet, have built a similar substrate cavity using its large C-terminal and flexible loop domains. BVMOs however seem to possess similar tunneling features and gatekeeper motifs like FMOs from clades I, III and IV. The principal differences in substrate profile seem to stem from the NADP-binding insertion and C-terminal domains that vary substantially across the phylogeny.

Gathering all evidence, it seems clear that two key elements govern FMOs physiological role: (i) protonation state of the FAD-based intermediate that resultantly mediates BV and N/S oxidations, and (ii) substrate channeling which orchestrates the range of potential substrates. The former is dictated by a concert of primary and secondary sphere residues and likely by the microenvironmental pH which varies across cellular compartments. Regardless of the protonation state, extensive studies on clade I and II FMOs suggests that the intermediate behaves like a cocked gun, primed for activity. This intermediate is postulated to be stabilized by the oxidized coenzyme, NADP^+^, that remains bound throughout catalysis [31]. This is likely commensurate with clades III and IV as BVMOs and NMOs also exhibit this phenomenon. Collectively, it seems clear that the main chemistry of FMOs is derived from the electrophilic mechanism. Between S and N oxygenations no clear substrate preferences can be traced or even predicted, as both reactions would essentially involve the same mechanism. Moreover, due to the nature of the catalytic mechanism itself, the substrate scope would be mainly dictated by the accessing tunnel, hence virtually any amine or sulfide able to reach the active site could be oxidized. Generalizing the substrate scope of FMOs performing BV oxidations is difficult as less members of this type have been characterized. The BV type of oxygenation is the result of a complex set of interactions among a catalytic network that involves not only active site residues, but also the nicotinamide molecule working as hydride donor and the flavin cofactor [31]. On the basis of the work by Bailleul, Yang and coauthors [40], it is reasonable to predict that other members of clade I FMOs, as some of the sequences from the deer tick (*Ixodes scapularis*) would be able to perform the same type of chemistry as they exhibit the same catalytic network. Interestingly, for the clade III members performing BV oxidations, the network is partially conserved but some completely new structural elements emerge. Therefore, it seems that BV arose by convergence mechanism in different clades from this enzyme family. This raises again the question; can this activity be considered as archetypal for FMOs? We suspect that indeed it might be the case, however still more extant FMO members should be experimentally characterized to make such a statement.

FMOs have been considered non-crucial enzymes for organisms. However, with the amount of experimental information gathered over the years, interpreted on the grounds of a comprehensive phylogeny as the one we provide, this notion should be challenged. FMOs are largely conserved among all living things and show an impressive degree of functional divergence. Likely, FMOs evolved to fulfill new ecological roles originated from changing environmental conditions, positively impacting on the species fitness.

## Outlook

For future work on the characterization of novel FMOs we provide here three main aspects to be considered:

1) Clades of FMOs are highly informative. In this regard, our tree is robust to the inclusion of more sequences. This was tested several times by adding sequences of known FMOs to the dataset and then re-inferring the tree. Sequences cluster where expected and that is coincident with the biochemical data available and structural (predicted) data. We suggest to initially performing a simple evolutionary analysis as the features for unknown enzymes can be disclosed.
2) The topology of FMO phylogeny encloses an inherent challenge to be considered: it does not follow the species tree. Therefore, particular phylogenies of isolated taxonomic groups and conclusions about their functional divergence/ancestral features should be avoided, instead protein clades should be considered.
3) The names coined to the groups in the family are ambiguous since functionalities seem to be more overleaped than expected. Therefore, we propose to stick to FMOs, NMOs and BVMOs as names due to historical reasons, but to introduce the term *tDNBD monooxygenases* coupled to *Group B monooxygenases*. This terminology provides insight regarding the evolution of functionality in this family.

## Methods

### Dataset construction

Sequences were collected among all phyla or classes from the three domains of life. To do that, up to three species with fully sequenced genomes according to NCBI genome information (lastly accessed in Nov 2022) were selected. When no species with full genomic data was available, those displaying at least the scaffold level (or contig if no other) were selected. Homology searches were performed using the protein sequences of the 5 FMO paralogs from *Homo sapiens*. All hits belonging to different paralogs (no isoforms/variants) from each species mined were collected into an initial dataset. Then, sequences were vetted for the presence of the FMO conserved motif FxGxxxHxxx(Y/F)(K/R) (and the absence of BVMOs fingerprints, GGxWxxxx(F/Y)P(G/M)xxxD and FxGxxxHxxxW(P/D)). Those sequences showing a partially conserved FMO fingerprint were also collected. In total 181 FMO sequences were included. As external groups sequences from BVMOs (38 in total, covering the three domains of life) and from NMOs (22 in total) were included.

Multiple sequence alignments were built with MAFFT v7 [54]. Single seqs insertions and deletions were manually trimmed.

### Phylogenetic inference

RaxML v8.2.10 was employed to build the phylogeny [55]. A MSA containing 254 sequences and 559 sites was employed. The substitution model and parameters were also computed by RaxML (LG+*F*, α= 1.91). Rapid bootstrap analysis was performed to compute the best scoring ML tree and 500 bootstraps were run. Transfer bootstrap expectation (TBE) values were calculated with BOOSTER online tool [56]. The obtained tree was midpoint rooted and the external groups, BVMOs and NMOs, were recovered as monophyletic. The tree was reinterred several times with the addition of experimentally characterized FMOs to test its robustness to the inclusion of more sequences and the topology recovered was always consistent.

### Structural analyses

All structural analyses were performed using UCSF Chimera [57]. Representative FMO proteins were first selected based on crystal structure availability before turning to deposited Alphafold2 predicted structures. The following crystal structures were analyzed: AncFMO5 (Synthetic construct, PDB:6SEK, clade I), AncFMO3–6 (Synthetic construct, PDB:6SE3, clade I) and mFMO (*Methylophaga aminisulfidivorans*, PDB:2VQ7, clade II). The following AlphaFold2 models were used for analysis: AtFMO (*Arabidopsis thaliana*, OAP18020, clade IV), TcFMO (*Trypanosoma cruzi*, XP_817,059, clade IV), AcFMO (*Acanthamoeba castellanii*, XP_004335213, clade I), YUCCA1 (*Arabidopsis thaliana*, OAP00458, clade III), FMOA (*Rhodococcus jostii*, WP_011593985, clade III), FMOF (*Rhodococcus jostii*, WP_011596698, clade III). Structure based MSAs were constructed using ESPript3 (https://espript.ibcp.fr/) [58].

## Funding sources

This work was funded by: European Union's Horizon 2020 Research and Innovation program under grant agreement No. 847675 COFUND project oLife (MLM).

## Declaration of Competing Interest

The authors declare the following financial interests/personal relationships which may be considered as potential competing interests:

Associate editor in BBA Advances, MLM

## Data Availability

All data is available in the SI

## Appendix Supplementary materials

Image, application 1
