# Supplementary material for: Investigating the biochemical signatures and physiological roles of the FMO family using molecular phylogeny
Source: BBA Adv. 2023 Nov 4;4:100108. doi: 10.1016/j.bbadva.2023.100108 (PMC10682829; doi:10.1016/j.bbadva.2023.100108)
Supplement: Supplementary file 1 [file mmc1.docx]

***Investigating the biochemical signatures and physiological roles of the FMO family using molecular phylogeny***

*Nicoll C.R.^1^ & Mascotti M.L^2,3^*

1. Department of Biology and Biotechnology Lazzaro Spallanzani, University of Pavia, Via Ferrata 9, 27100, Pavia, Italy

2. Molecular Enzymology, Groningen Biomolecular Sciences and Biotechnology Institute, University of Groningen, Nijenborgh 4, 9747, AG Groningen, The Netherlands

3. IMIBIO-SL CONICET, Facultad de Química Bioquímica y Farmacia, Universidad Nacional de San Luis, Ejército de los Andes 950, D5700HHW, San Luis, Argentina

**Supplementary Information**

**List of contents**

- **Table S1.** Species vetted for FMOs presence
- **Fig. S1.** FMOs clade distribution in the Eukarya domain
- **Fig. S2.** FMOs fully annotated phylogeny
- **Table S2.** List of representative FMOs experimentally characterized to date
- **Fig. S3.** Structure-based multiple sequence alignment of representative FMOs
- **Fig. S4.** Structural topology of BVMOs and NMOs
- **Fig. S5.** Gate-keeping features of BVMOs and NMOs
- **Fig.** **S6.** Active site features for BVMOs and NMOs.

**Table S1.** List of species vetted for the presence of FMOs and accession codes (GenBank or Uniprot) of selected paralogs.

| **Phyla** | **Full genome** | **Species** | **FMO name** | **Accession** |
| --- | --- | --- | --- | --- |
| Preaxostyla | - | *Monocercomonoides exilis* |  | X |
| Fornicata | yes | *Spironucleus salmonicida* |  | X |
|  | yes | *Giardia intestinalis* |  | X |
| Chlorophyta | yes | *Chlorella* sp |  | XP_005849556 |
|  | yes | *Chlamydomonas* sp |  | KAG2443029 |
| Streptophyta | yes | *Arabidopsis thaliana* |  | OAO96107 |
|  |  |  |  | NP_172680 |
|  |  |  |  | NP_001323033 |
|  |  |  | FMO1 | OAP18020 |
|  |  |  |  | NP_199331 |
|  |  |  |  | NP_196397 |
|  |  |  | FMO GSOX3 | OAP12395 |
|  |  |  | FMO GSOX1 | OAP12743 |
|  |  |  |  | NP_001323101 |
|  |  |  |  | CAD5316092 |
|  |  |  | YUC6 | OAO91774 |
|  |  |  | YUC1 | OAP00458 |
|  |  |  |  | NP_850808 |
|  |  |  | YUC9 | NP_171914 |
|  |  |  | YUC11 | OAP16106 |
|  |  |  |  | CAD5331110 |
|  |  |  |  | NP_001322976 |
|  |  |  | FMO GSOX4 | NP_564797 |
|  |  |  |  | NP_176523 |
|  |  |  | FMO GSOX5 | NP_001077522 |
|  |  |  | YUC2 | OAO97792 |
|  |  |  | YUC3 | NP_171955 |
|  |  |  | YUC7 | NP_180881 |
|  | yes | *Oryza sativa* | FMO_GSOX8 | XP_015645788 |
|  |  |  | FMO_GSOX9 | XP_015642183 |
|  |  |  | FMO_GSOX9 | XP_015624769 |
|  |  |  | YUCCA9 | XP_015619266 |
|  |  |  | FMO4 | BAD61442 |
|  |  |  | FMO_GSOX4 | XP_015612837 |
|  |  |  | YUCCA11 | XP_015619543 |
|  |  |  | YUCCA10 | XP_015616152 |
|  |  |  | YUCCA10 | XP_015629696 |
|  |  |  | YUCCA3 | BAA88198 |
|  |  |  | YUCCA2 | XP_025876562 |
|  |  |  | YUCCA2 | XP_015638961 |
|  |  |  | YUCCA5 | XP_015634157 |
|  |  |  | YUCCA4 | XP_015643006 |
|  |  |  | YUCCA1 | XP_015631023 |
|  |  |  | FMO1 | BAD89476 |
|  |  |  | FMO1 | XP_015629366 |
|  |  |  | FMO1 | XP_015612285 |
|  |  |  | FMO1 | XP_015633535 |
|  |  |  | FMO1 | XP_015611435 |
|  |  |  | FMO2 | XP_025882417 |
|  |  |  | YUCCA2 | XP_015632133 |
|  | yes | *Physcomitrium patens* | FMO_GSOX4 | XP_024389140 |
|  |  |  | YUCCA6 | XP_024388169 |
|  |  |  | YUCCA9 | XP_024369309 |
|  |  |  | YUCCA5 | XP_024372648 |
| Rhodophyta | yes | *Galdieria sulphuraria* |  | XP_005703989 |
|  | yes | *Cyanidioschyzon merolae* |  | XP_005535933 |
| Porifera | yes | *Amphimedon queenslandica* |  | XP_003386831 |
|  |  |  |  | XP_003386180 |
|  | yes | *Chondrosia reniformis* |  | X |
| Hemichordata | yes | *Saccoglossus kowalevskii* |  | XP_002740302 |
|  |  |  |  | XP_006818115 |
|  |  |  |  | XP_002734588 |
|  |  |  |  | XP_002734589 |
|  |  |  |  | XP_002735404 |
| Echinodermata | yes | *Heliocidaris erythrogramma* |  | X |
| Xenacoelomorpha | yes | *Hofstenia miamia* |  | X |
| Chordata | yes | *Homo sapiens* | FMO1 | Q01740 |
|  |  |  | FMO2 | Q99518 |
|  |  |  | FMO3 | Q53FW5 |
|  |  |  | FMO4 | XP_005245102 |
|  |  |  | FMO5 | P49326 |
|  |  |  | FMO6 | O60774 |
|  | yes | *Xenopus laevis* |  | A0A1L8GN41 |
|  |  |  |  | NP_00108387 |
|  |  |  | FMO2 | XP_018114110 |
|  |  |  | FMO3 | XP_018112304 |
|  |  |  | FMO5 | XP_018114114 |
|  | yes | *Cuculus canorus* | FMO5 | XP_009563770 |
|  |  |  | FMO2 | XP_009558179 |
|  |  |  | FMO3 | XP_009558178 |
|  |  |  | FMO | XP_009558155 |
|  | yes | *Salmo trutta* | FMO | XP_029563292 |
| Tardigrada | yes | *Hypsibius exemplaris* |  | OWA53256 |
|  |  |  |  | OQV24618 |
|  |  |  |  | OQV20574 |
|  |  |  |  | OWA51249 |
|  |  |  |  | OWA51252 |
|  |  |  |  | OQV15017 |
| Onychophora | - | *Euperipatoides rowelli* |  | X |
| Arthropoda | yes | *Ixodes scapularis* |  | XP_029838655 |
|  |  |  |  | XP_040357517 |
|  |  |  |  | XP_029843021 |
|  |  |  |  | XP_040066569 |
|  |  |  |  | XP_040077570 |
|  |  |  |  | XP_042142250 |
|  |  |  |  | XP_040062451 |
|  |  |  |  | XP_040068438 |
|  |  |  |  | XP_040062454 |
|  |  |  |  | XP_029838652 |
|  |  |  |  | XP_002403130 |
|  |  |  |  | XP_042147279 |
|  |  |  |  | XP_040064364 |
|  |  |  |  | XP_002413027 |
|  |  |  |  | XP_042145270 |
|  | yes | *Drosophila melanogaster* |  | NP_610217.1 |
|  |  |  |  | NP_611859 |
| Rotifera | yes | *Adineta vaga* |  | UJR27596 |
|  |  |  |  | UJR37611 |
|  |  |  |  | UJR17555 |
|  |  |  |  | UJR28242 |
|  |  | *Brachionus plicatilis* |  | RNA19017 |
| Entoprocta | - |  |  |  |
| Annelida | yes | *Lumbricus rubellus* |  | X |
|  | yes | *Piscicola geometra* |  | X |
| Phoronida | - | *Phoronis australis* |  | X |
| Brachiopoda | - | *Lingula anatina* |  | XP_013400166 |
|  |  |  |  | XP_013413360 |
|  |  |  |  | XP_013400134 |
|  |  |  |  | XP_013410014 |
|  |  |  |  | XP_013392145 |
|  |  |  |  | XP_013388858 |
|  |  |  |  | XP_013410775 |
|  |  |  |  | XP_013404525 |
| Mollusca | yes | *Argopecten irradians* |  | X |
|  | yes | *Haliotis cracherodii* |  | X |
| Nematoda | yes | *Caenorhabditis elegans* |  | NP_501968 |
|  |  |  |  | NP_503352 |
|  |  |  |  | NP_501972 |
|  |  |  |  | NP_492038 |
|  |  |  |  | NP_491510 |
|  | yes | *Trichinella spiralis* |  | XP_003375540 |
| Platyhelminthes | yes | *Schmidtea mediterranea* |  | X |
|  | yes | *Schistosoma bovis* |  | X |
| Bryozoa | yes | *Cryptosula pallasiana* |  | X |
|  | yes | *Bugulina stolonifera* |  | X |
| Sipuncula | - |  |  |  |
| Chaetognatha | - |  |  |  |
| Cnidaria | yes | *Nematostella vectensis* |  | XP_001624119 |
|  |  |  |  | EDO41869 |
|  |  |  |  | XP_032236126 |
|  |  |  |  | EDO37068 |
|  | yes | *Clytia hemisphaerica* |  | X |
| Ctenophora | yes | *Pleurobrachia bachei* |  | X |
| Cryptomycota | yes | *Rozella allomycis* |  | X |
| Basidiomycota |  | *Cryptococcus neoformans* |  | OWZ32034 |
|  | yes | *Puccinia striiformis* |  | KAI9615107 |
|  |  |  |  | XP_047800203 |
| Ascomycota | yes | *Aspergillus fumigatus* |  | KAF4277179 |
|  |  |  |  | KAH3019204 |
|  |  |  |  | KAH1474772 |
|  | yes | *Yarrowia lipolytica* |  | RDW33055 |
| Zoopagomycota | yes | *Basidiobolus meristosporus* |  | ORX86866 |
|  |  |  |  | ORX90129 |
|  |  |  |  | ORY06269 |
|  |  |  |  | ORX91003 |
| Chytridiomycota | yes | *Batrachochytrium dendrobatidis* |  | OAJ35849 |
| Blastocladiomycota | - | *Catenaria anguillulae* |  | X |
| Microsporidia | yes | *Encephalitozoon cuniculi* |  | X |
|  | yes | *Nematocida parisii* |  | X |
| Haptophyta | yes | *Emiliania huxleyi* |  | XP_005765261 |
|  |  |  |  | XP_005780972 |
|  |  |  |  | XP_005787724 |
|  |  |  |  | XP_005756829 |
|  | yes | *Diacronema lutheri* |  | KAG8466522 |
|  |  |  |  | KAG8467582 |
| Bacillariophyta | yes | *Phaeodactylum tricornutum* |  | XP_002180330 |
|  | yes | *Fragilariopsis cylindrus* |  | OEU17181 |
| Oomycota | yes | *Phytophthora infestans* |  | XP_002999115 |
|  | yes | *Plasmopara halstedii* |  | XP_024578342 |
| Cercozoa | yes | *Bigelowiella natans* |  | X |
|  | yes | *Paulinella micropora* |  | X |
| Foraminifera | - | *Reticulomyxa filosa* |  | X |
| Endomyxa | - | *Haplosporidium sp* |  | X |
| Ciliophora | - | *Isotricha* |  | X |
| Perkinsozoa | - | *Perkinsus marinus* |  | X |
| Apicomplexa | yes | *Plasmodium falciparum* |  | X |
|  | yes | *Toxoplasma gondii* |  | X |
| Heterolobosea | yes | *Tetramitus* sp |  | X |
|  | yes | *Willaertia magna* |  | X |
| Euglenozoa | yes | *Trypanosoma cruzi* | TcFMO | XP_817059 |
|  | yes | *Leishmania major* |  | XP_001681924 |
| Tubulinea | x | *Vermamoeba vermiformis* |  | X |
| Discosea | yes | *Acanthamoeba castellanii* |  | XP_004341272 |
|  |  |  |  | XP_004337495 |
|  |  |  |  | XP_004335213 |
|  |  |  |  | XP_004339335 |
|  | yes | *Balamuthia mandrillaris* |  | X |


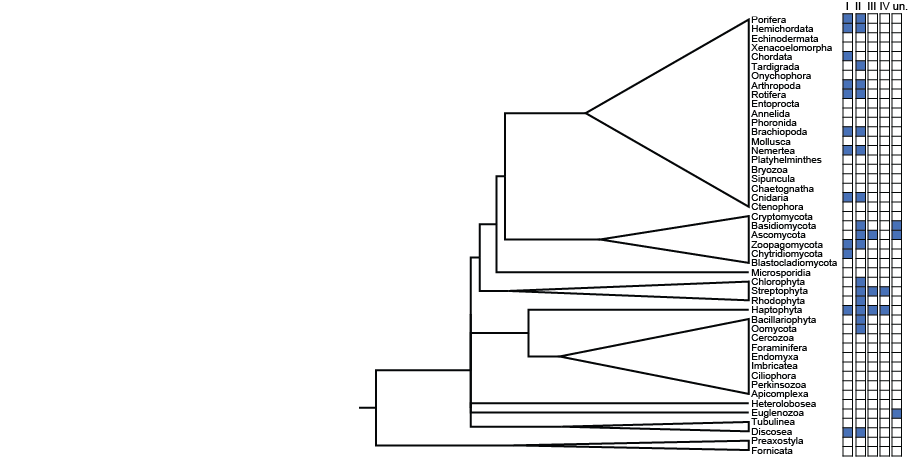


**Fig. S1. FMOs clade distribution in the Eukarya domain.** The filled boxes indicate the presence and empty absence. Clades are defined in the main text.


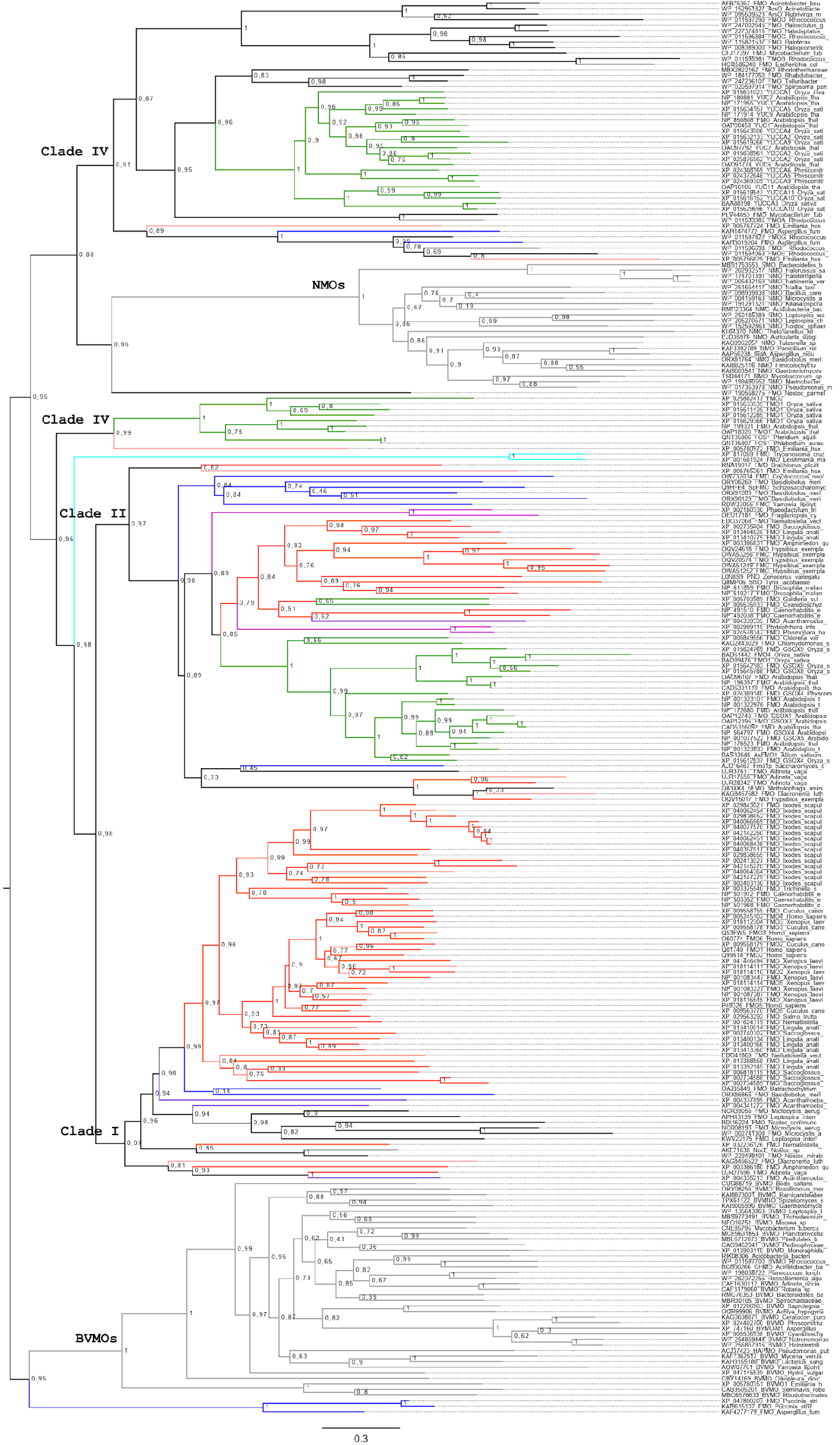


**Fig. S2. FMOs fully annotated FMOs phylogeny.** The tree is presented as a midpoint rooted. It was obtained with RaxML (500 BS) and support values obtained with BOOSTER (TBE) are shown above the branches. Branches are colored according to the taxonomy as follows: Bacteria (black), Chlorophyta, Streptophyta and Rhodophyta (green), Porifera, Hemichordata, Chordata, Tardigrada, Arthropoda, Rotifera, Brachiopoda, Nematoda and Cnidaria (red), Basidiomycota, Ascomycota, Zoopagomycota and Chytridiomycota (blue), Haptophyta (pink), Bacillariophyta and Oomycota (magenta), Euglenozoa (cyan) and Discosea (violet). NMO and BVMO clades are shown with gray branches. The scale bar indicates substitutions per site.

**Table S2.** List of representative FMOs experimentally characterized to date.

| **Clade** | **Genbank/**  **Uniprot** | **Name** | **Organism** | **PDB** | **Length (aa)** | **Activity** | **Substrates** | **Physiological role** | **Ref.** |
| --- | --- | --- | --- | --- | --- | --- | --- | --- | --- |
| I | UZZ64692 | mAncFMO3-6 | ancestor mammals | 6SE3 | 532 | S oxidation | benzylphenyl sulfide, MPTS, thioanisole | Detoxification metabolism, regulation of cellular stress resistance and regulation of central carbon metabolism | ^1-4^ |
|  |  |  |  |  |  | N oxidation | tamoxifen, benzydamine, methimazole, trimethylamine |  |  |
|  | UZZ64693 | mAncFMO5 | ancestor mammals | 6SEK | 533 | BV oxidation | alkyl, cycloalkyl and alkylaryl ketones |  |  |
| II | WP_007144064 (Q83XK4) | mFMO/ bFMO | *Methylophaga aminisulfidivorans* | 2VQ7, 2XVH, 2XVJ | 456 | N oxidation | trimethylamine, methimazole, nicotine, N,N-dimethylaniline, indole |  | ^5-7^ |
|  | CBX26645 (L0N8S9) | PNO | *Zonocerus variegatus* | 5NMW | 413 | N oxidation | pyrrolizidine alkaloids | Detoxification metabolism | ^8^ |
|  | Q8MP06 | SNO | *Tyria jacobaeae* |  | 456 | N oxidation | pyrrolizidine alkaloids | Detoxification metabolism | ^9^ |
|  | OAP12743 (A0A178W5U2) | FMO-GSOX1 | *Arabidopsis thaliana* |  | 459 | S oxidation | methylthioalkyl glucosinate | Secondary metabolism biosynthesis | ^10^ |
|  | AJU16467 (P38866) | FMO1p/ yFMO | *Saccharomyces cerevisiae* |  | 432 | S oxidation | cysteine, cystamine, glutathion | Redox regulation | ^11,12^ |
|  | Q9HFE4 | SpFMO | *Schizosaccharomyces pombe* | 2GV8 | 447 | S oxidation | methimazole |  | ^13^ |
|  | BAS32646 (A0A0M4U3V7) | AsFMO1 | *Allium sativum* | 6WPU | 457 | S oxidation | allyl mercaptan, cysteine, N-acetyl cysteine | Secondary metabolism biosynthesis | ^14,15^ |
| III | WP_011596698 (Q0S8V1) | FMOF | *Rhodococcus jostii* |  | 607 | BV oxidation | phenylacetone, bicycloheptanone, cyclobutanone, fused cyclobutanones, norcamphor |  | ^16-18^ |
|  |  |  |  |  |  | S oxidation | thioanisole (low), benzylethylsulfide (low) |  |  |
|  | XP_015631023 (A0A0E0GH52) | YUCCA 1/ OsFMO | *Oryza nivara* |  | 435 | BV oxidation | indole-3-piruvate (to indol acetic acid) | Secondary metabolism biosynthesis | ^19^ |
|  | OAP00458 (A0A178V499) | YUCCA1 | *Arabidopsis thaliana* |  | 414 | N-oxidation | tryptamine |  | ^20^ |
|  | WP_011593985 (A0A1H4IID2) | FMOA | *Rhodococcus jostii* |  | 375 | S oxidation | thioanisole (low) |  | ^16^ |
|  | WP_011595981 (Q0SBE9) | FMOB | *Rhodococcus jostii* |  | 418 | S oxidation | thioanisole (low) |  |  |
| IV | OAP18020 (A0A178WI39) | AtFMO1 | *Arabidopsis thaliana* |  | 530 | N oxidation | pipecolic acid | Systemic acquired resistance (SAR) | ^21^ |
|  | QNT35807 (A0A7H1KPE3) | FOS1 | *Phlebodium aureum* |  | 543 | N oxidation | L-phenylalanine | Secondary metabolism biosynthesis | ^22^ |
|  | QNT35806 (A0A7H1KPE2) | FOS1 | *Pteridium aquilinum* |  | 543 |  |  |  |  |
| unassigned | XP_817059 (Q4DRR6) | TcFMO | *Trypanosoma cruzi* |  | 550 | N-oxidation | dimethylaniline | Detoxification metabolism | ^23^ |


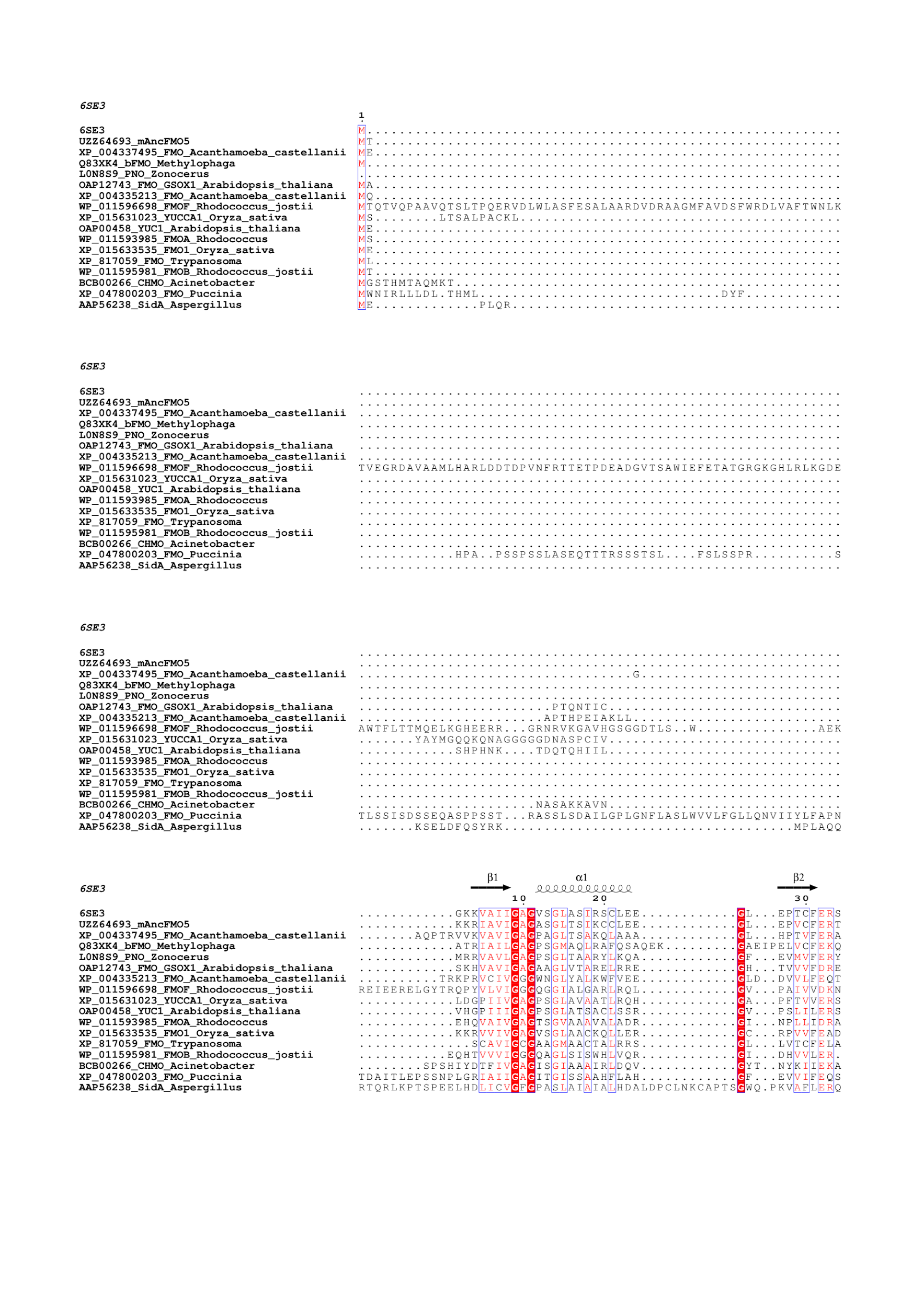

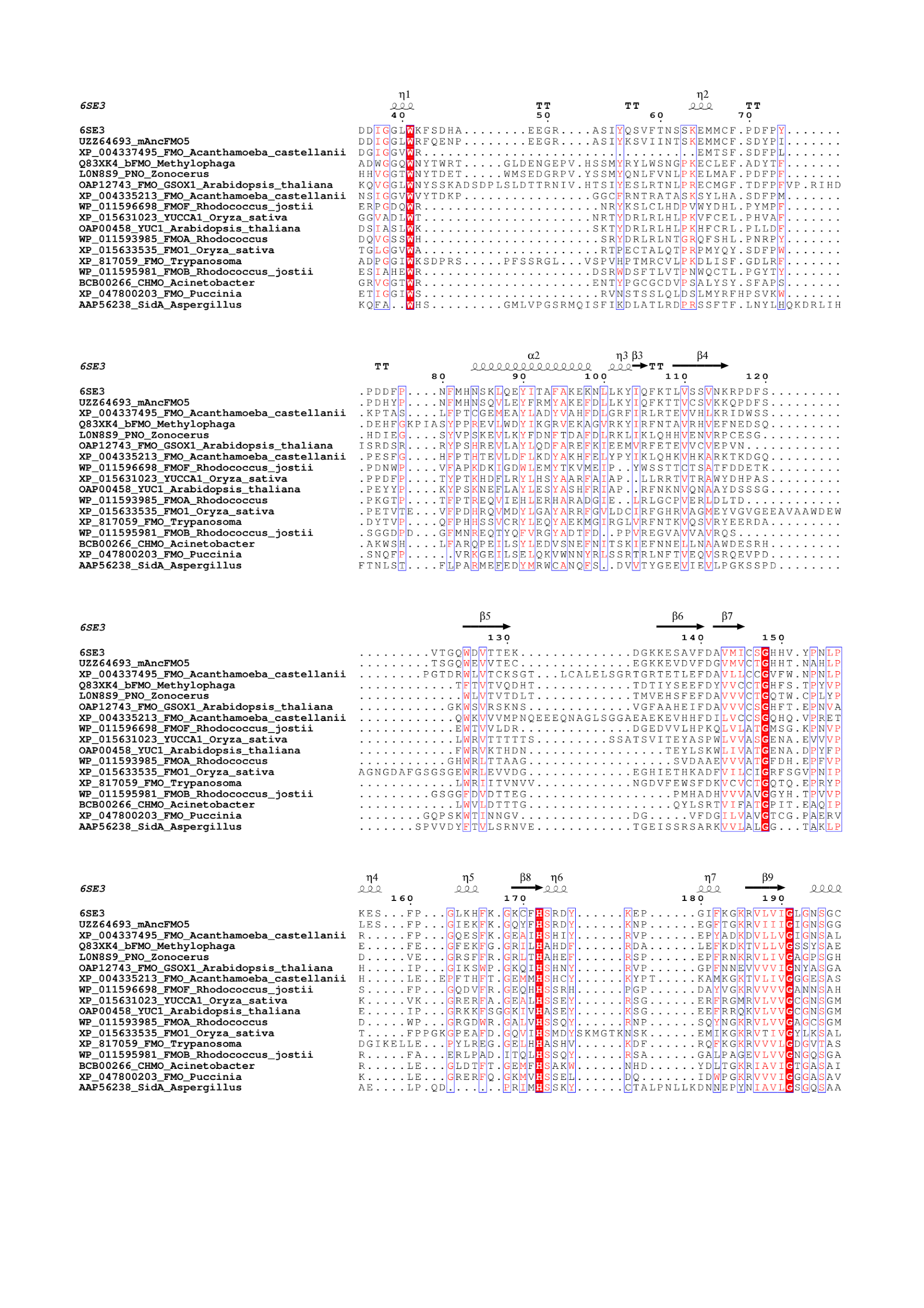

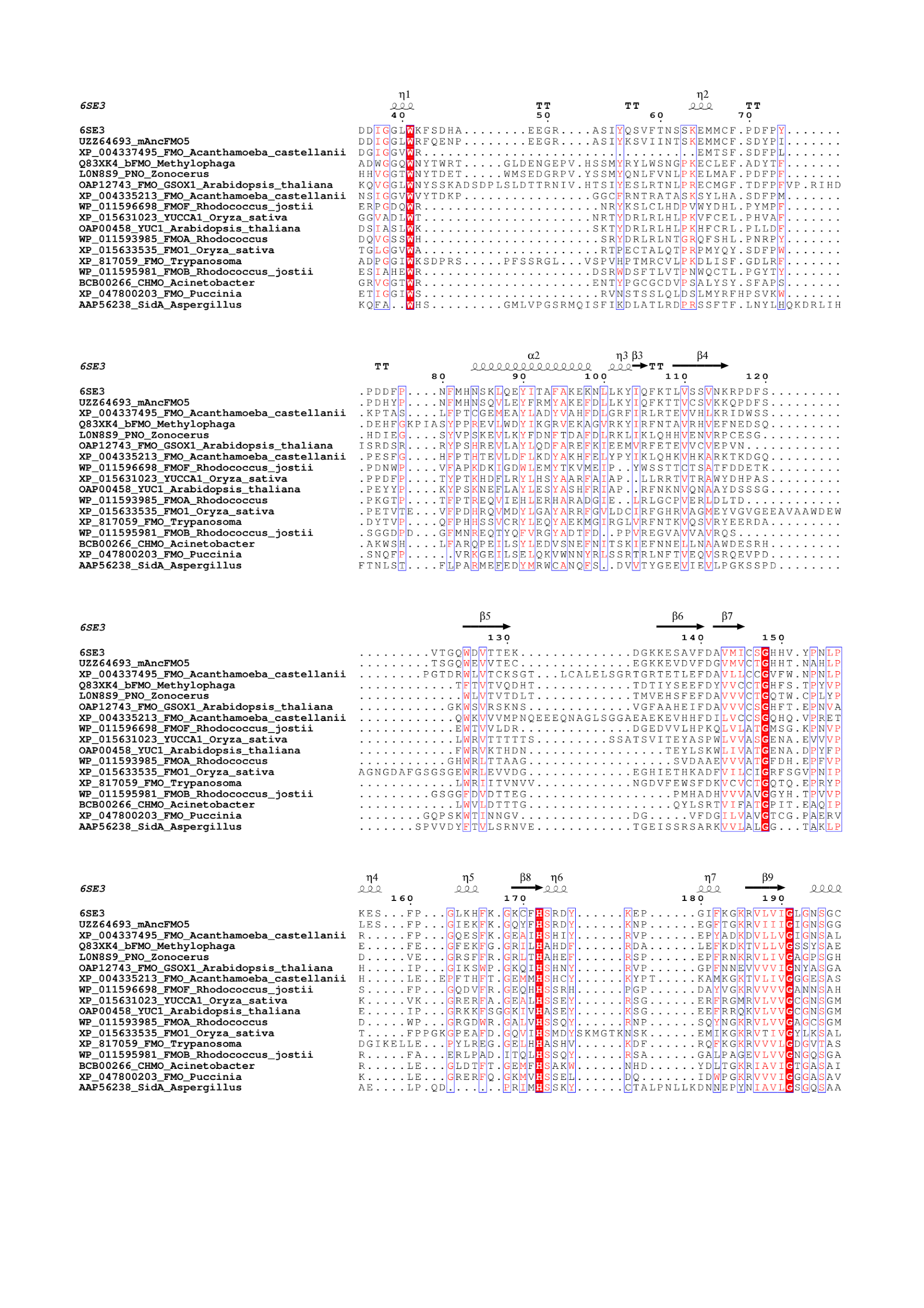

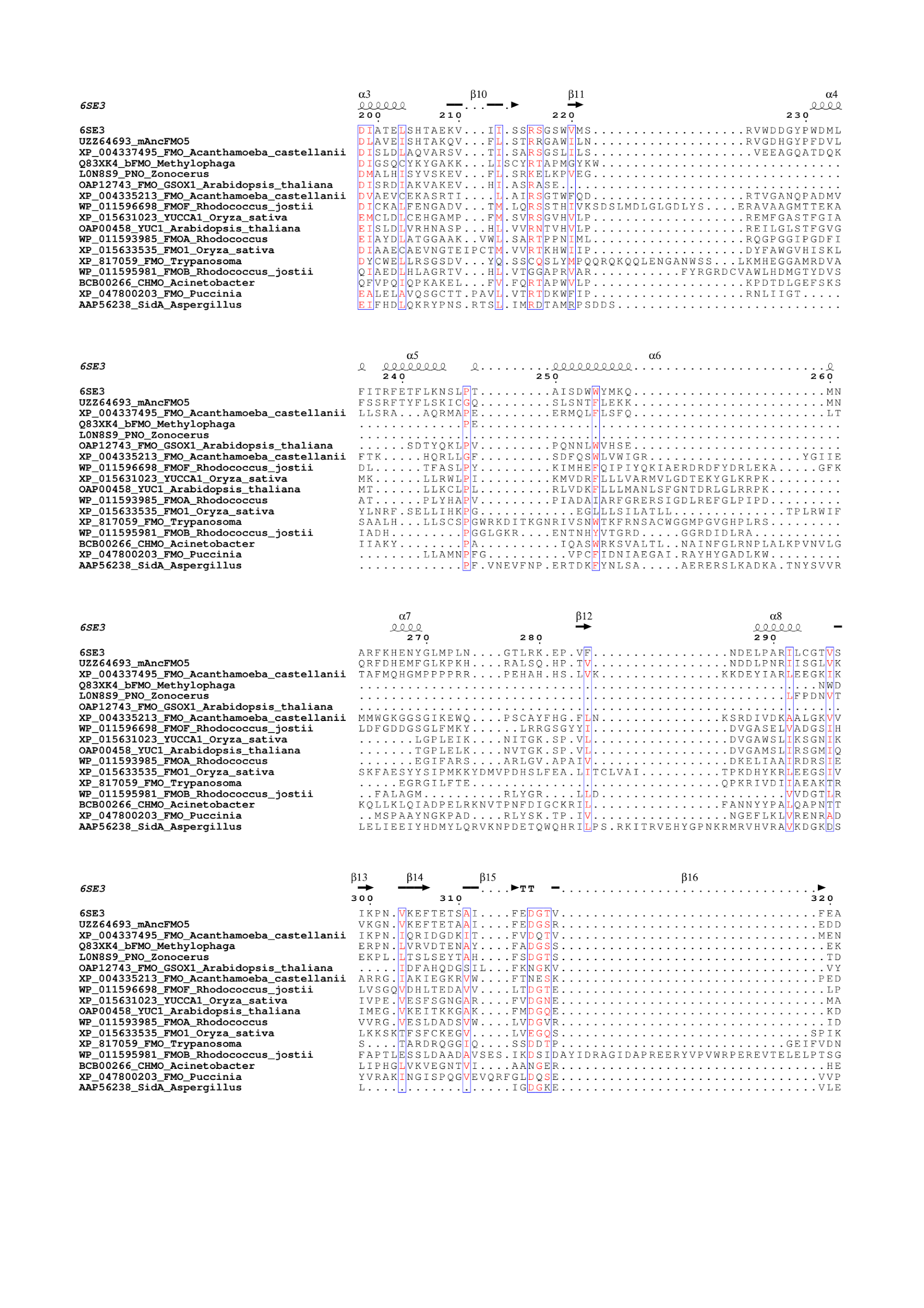

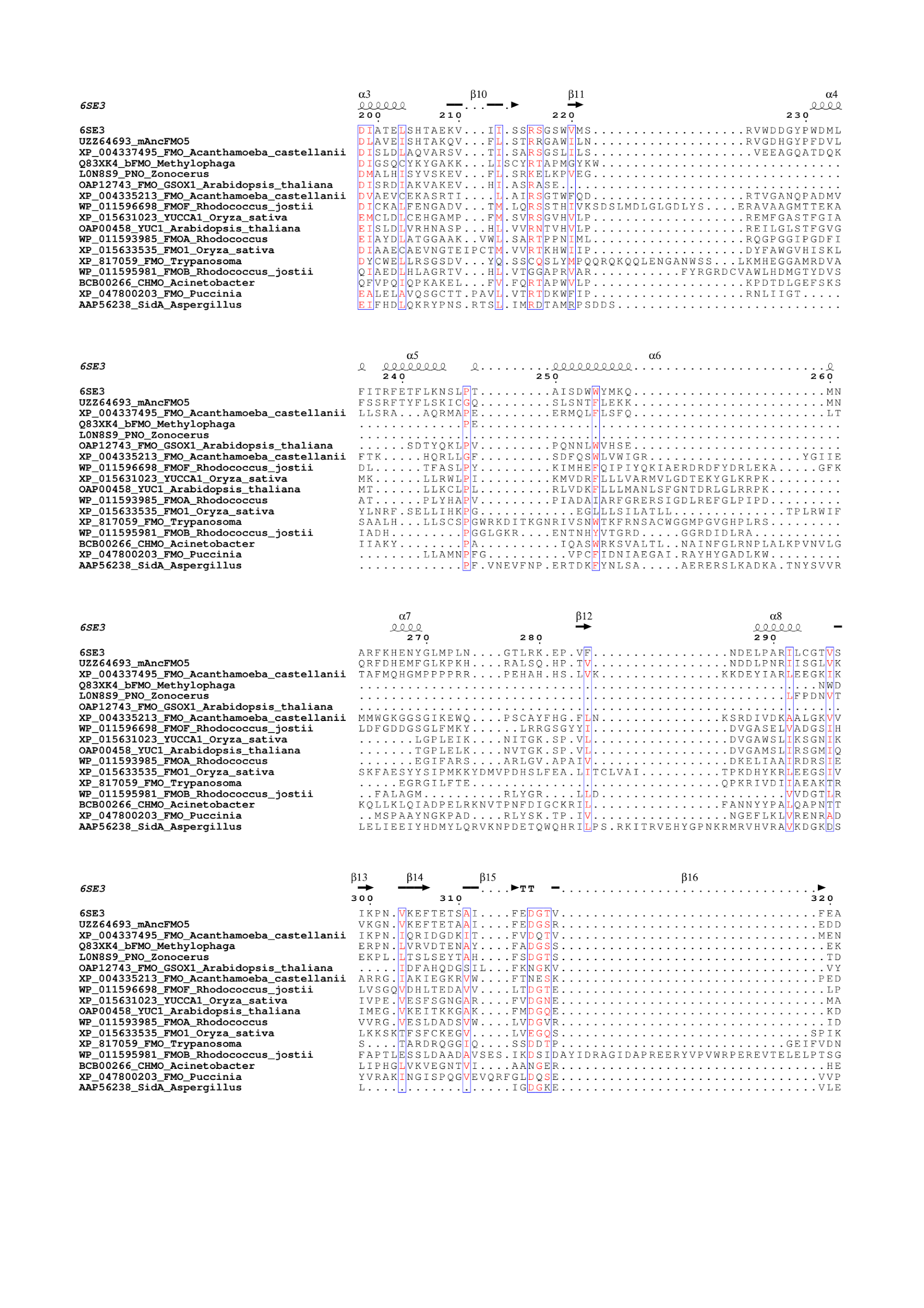

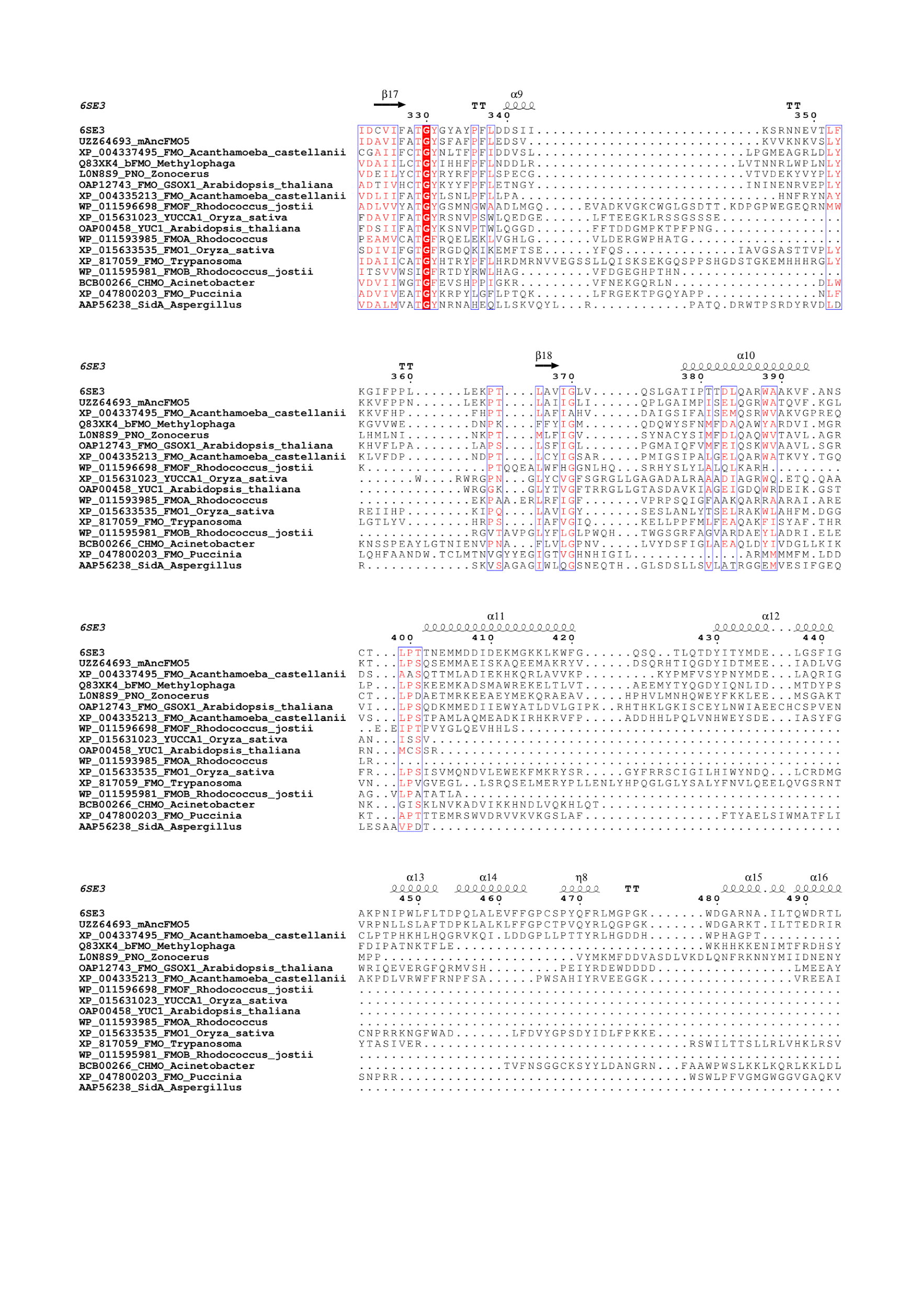

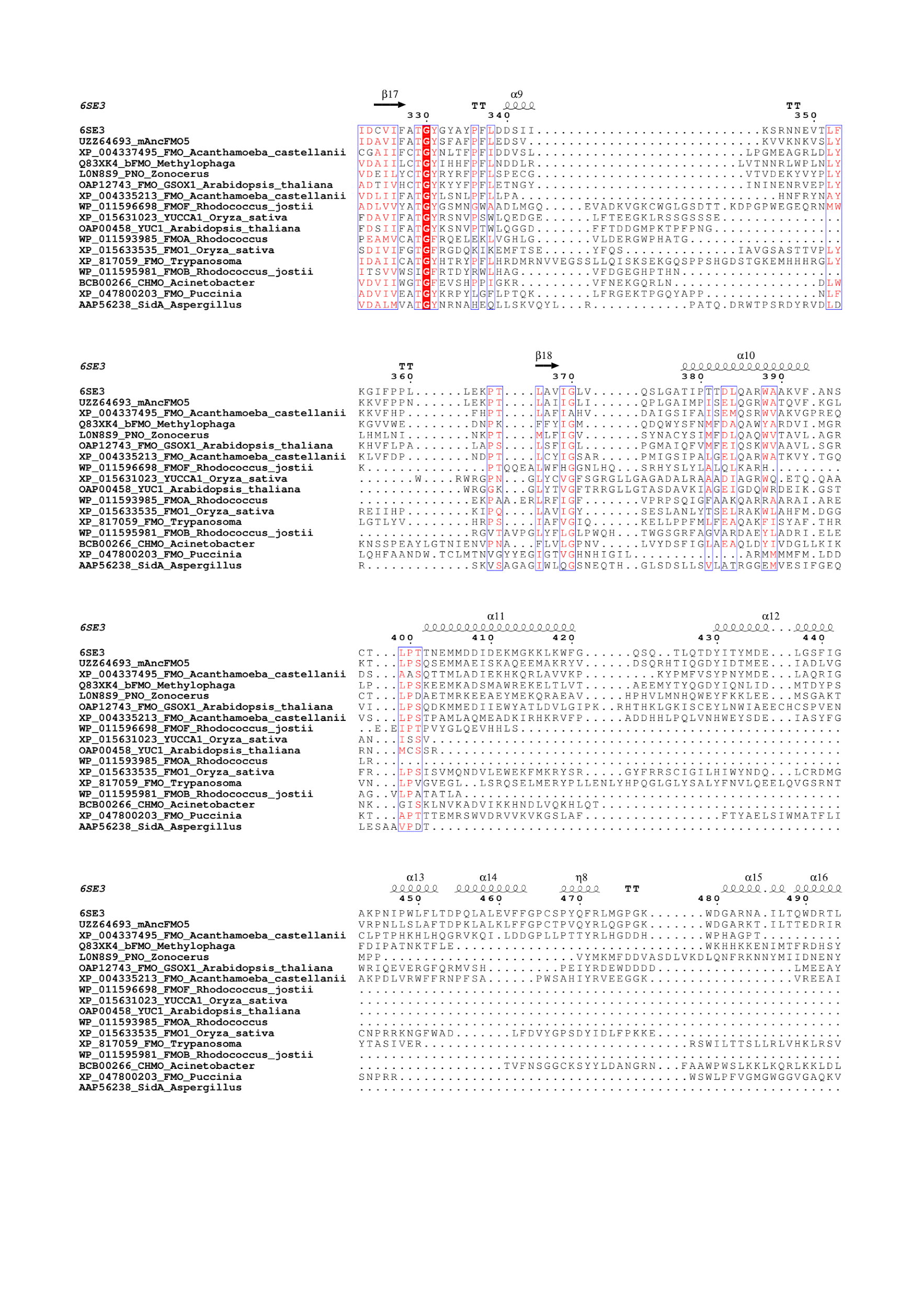

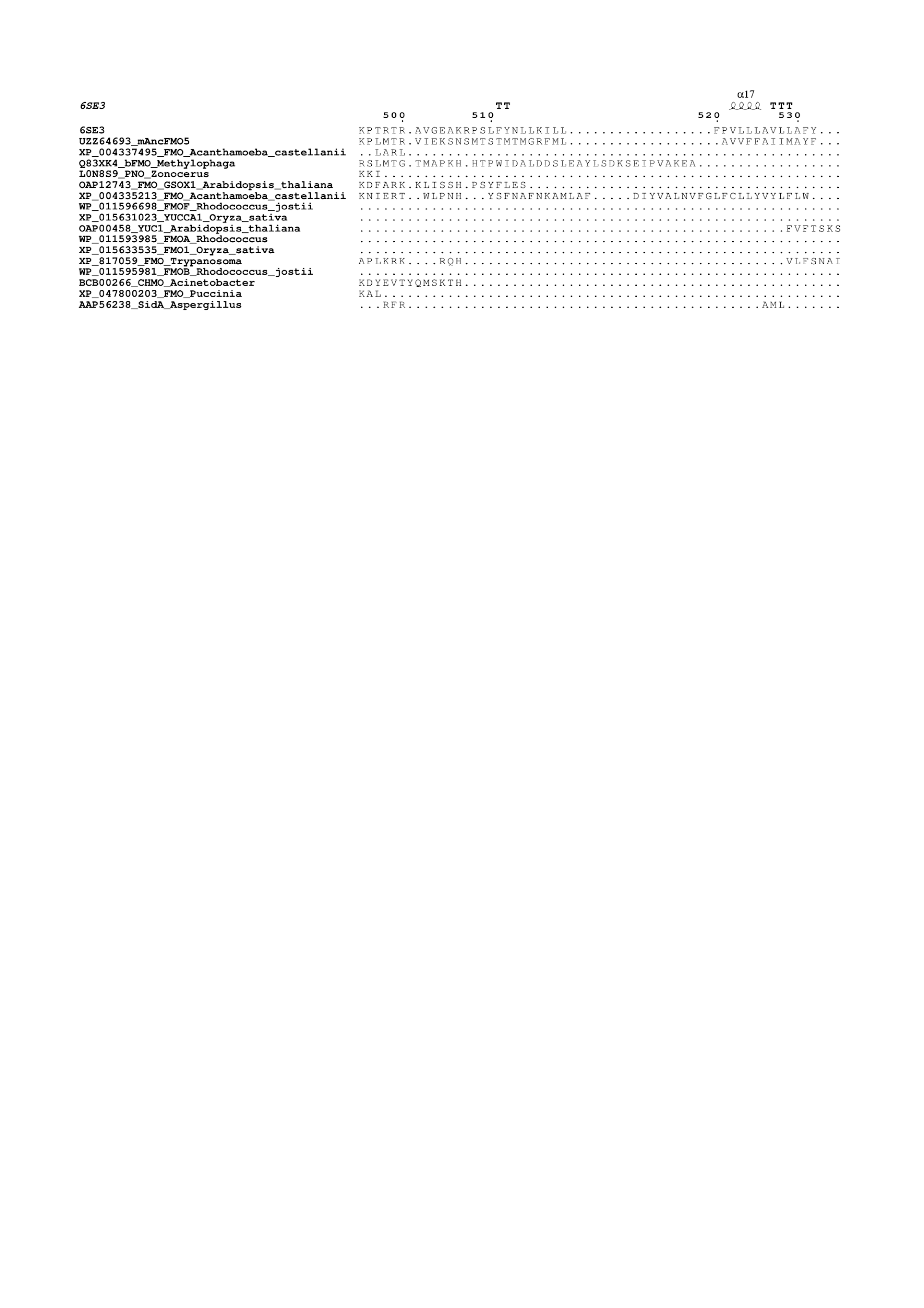


**Fig. S3. Structure-based multiple sequence alignment of representative FMOs from each clade.** The MSA was obtained with ESPript3. A BVMO sequence (BCB00266, CHMO from *Acinetobacter baumannii*) and a NMO sequence (AAP56238, SidA from *Aspergillus nidulans*) were also included.


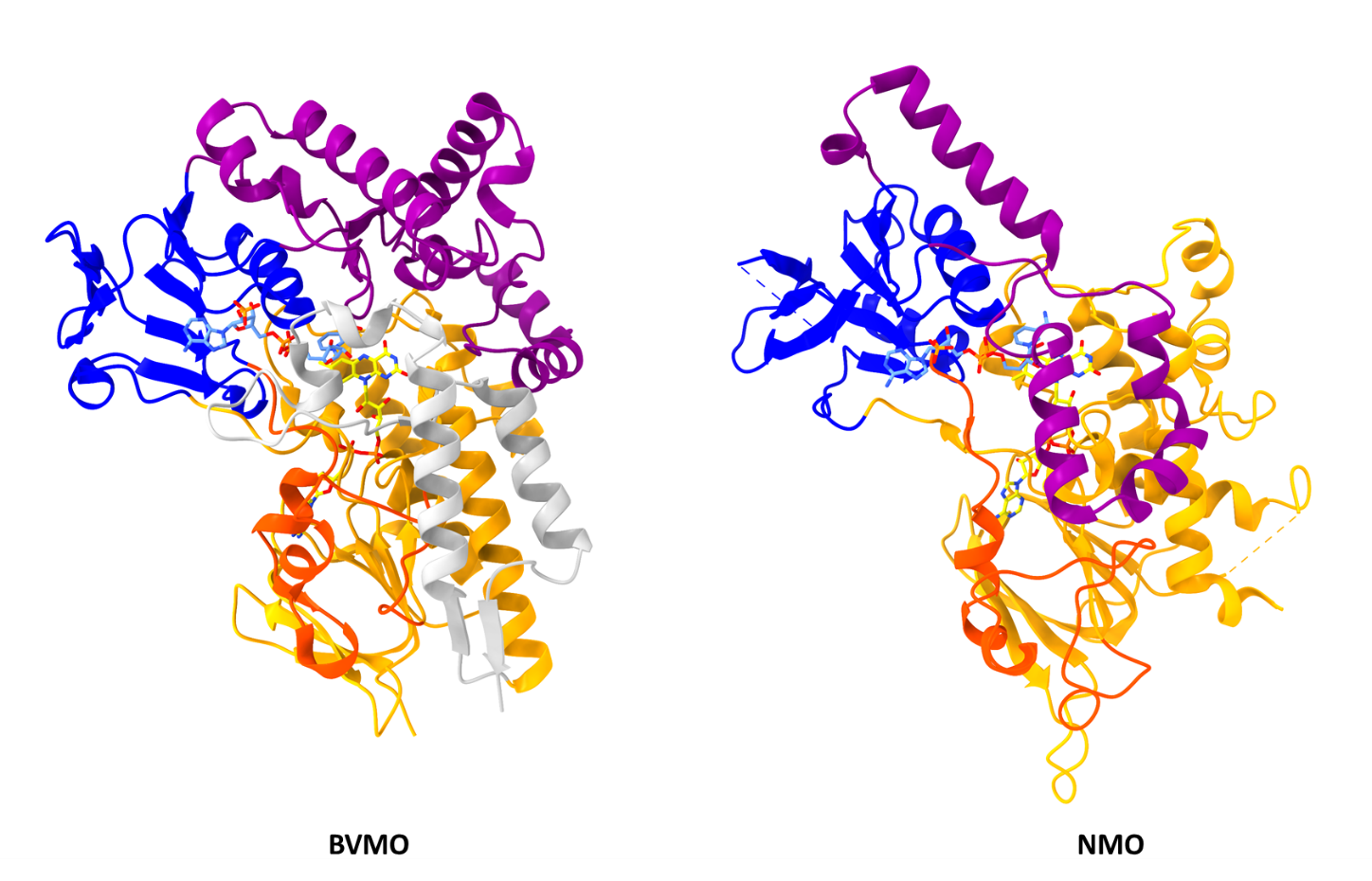


**Fig. S4. Structural topology of BVMOs and NMOs**. Dissection of the Class B structures into its individual domains. The highly conserved FAD- and NADP-binding domains that collectively illustrate the paired-Rossmann fold are shown in orange and blue, respectively. The NADP-binding domain insertion, the flexible loop domain and the C-terminal domain are shown in purple, dark orange and dark grey, respectively. The representative BVMO structure is from *Thermocrispum municipale* (PDB:5m10), referred to as CHMO^24^. The representative NMO structure is from *Aspergillus fumigatus Af293* (PDB: 6x0i), referred to as SidA^25^. Cofactors FAD and NADP^+^ are shown in yellow and cornflower blue, respectively.


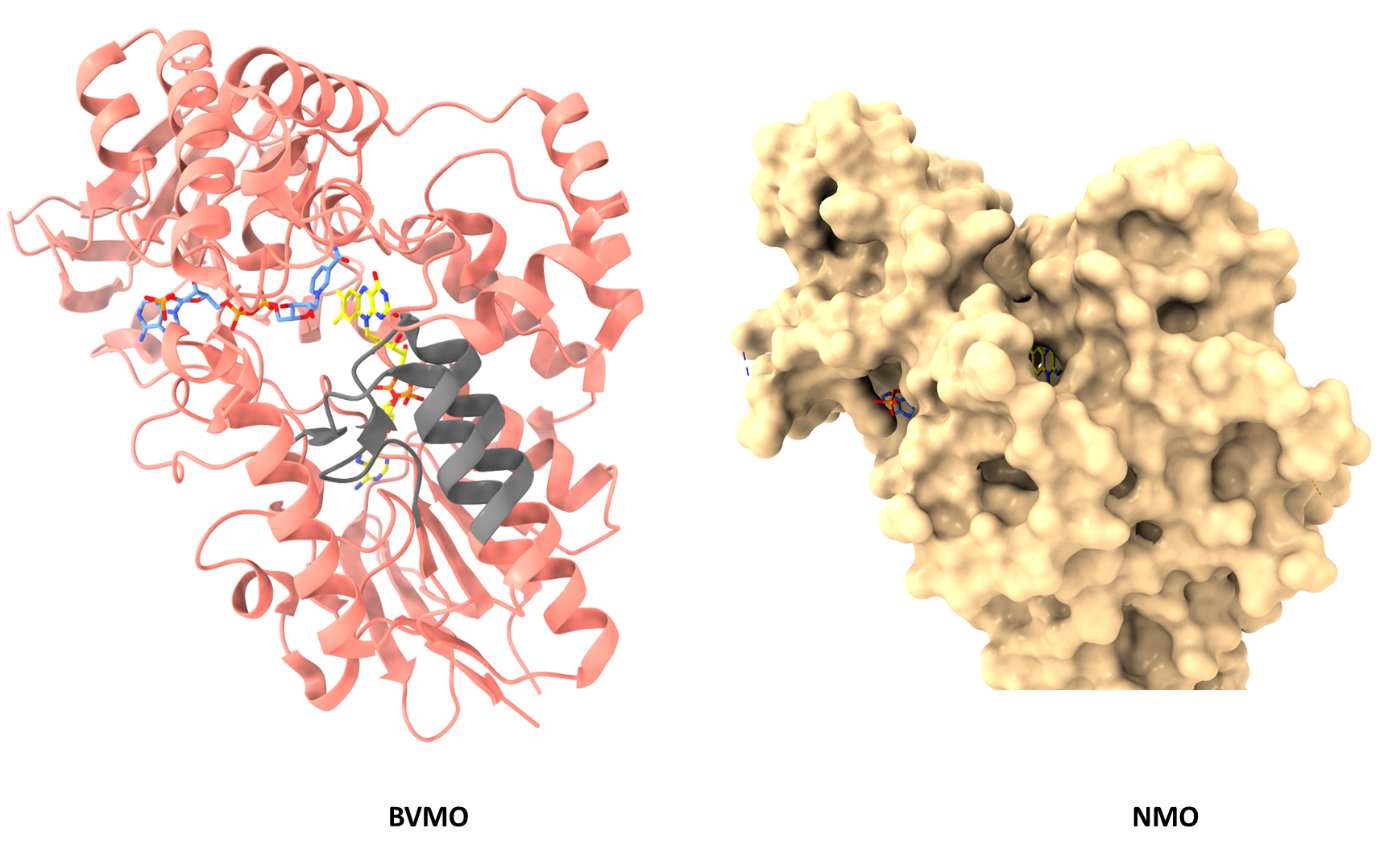


**Fig. S5. Gate-keeping features of BVMOs and NMOs**. Left, structural topology of a BVMO with its gate-keeping motif shown in dark gray. Right, surface representation of an NMO with its large substrate cavity indicated with an arrow. The representative BVMO structure is from *Thermocrispum municipale* (PDB:5m10), referred to as CHMO. The representative NMO structure is from *Aspergillus fumigatus Af293* (PDB: 6x0i), referred to as SidA. Cofactors FAD and NADP^+^ are shown in yellow and cornflower blue, respectively.


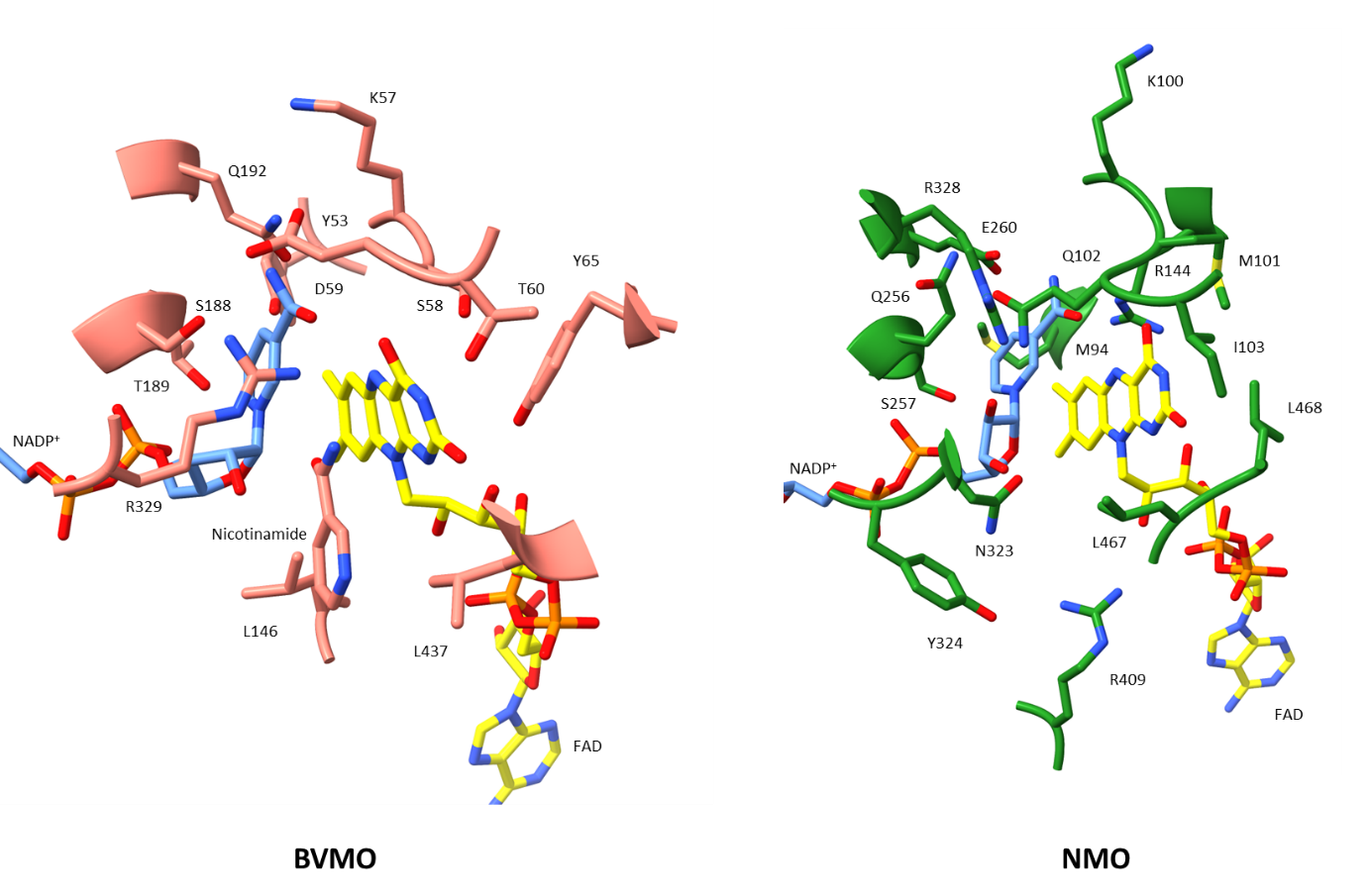
**Fig. S6. Active site features for BVMOs and NMOs**. Left, active site residues are shown for the representative BVMO structure from *Thermocrispum municipale* (PDB:5m10), referred to as CHMO. A molecule of nicotinamide is labelled. Right, active site residues are shown for the representative NMO structure from *Aspergillus fumigatus Af293* (PDB: 6x0i), referred to as SidA. Cofactors FAD and NADP^+^ are shown in yellow and cornflower blue, respectively.

**References**

1. Nicoll, C.R. et al. Ancestral-sequence reconstruction unveils the structural basis of function in mammalian FMOs. *Nature Structural & Molecular Biology* **27**, 14-24 (2020).

2. Bailleul, G., Nicoll, C.R., Mascotti, M.L., Mattevi, A. & Fraaije, M.W. Ancestral reconstruction of mammalian FMO1 enables structural determination, revealing unique features that explain its catalytic properties. *Journal of Biological Chemistry* **296**, 100221 (2021).

3. Huang, S., Howington, M.B., Dobry, C.J., Evans, C.R. & Leiser, S.F. Flavin-Containing Monooxygenases Are Conserved Regulators of Stress Resistance and Metabolism. **9**(2021).

4. Fiorentini, F. et al. Biocatalytic Characterization of Human FMO5: Unearthing Baeyer-Villiger Reactions in Humans. *ACS Chem Biol* **11**, 1039-48 (2016).

5. Alfieri, A., Malito, E., Orru, R., Fraaije, M.W. & Mattevi, A. Revealing the moonlighting role of NADP in the structure of a flavin-containing monooxygenase. *Proc Natl Acad Sci U S A* **105**, 6572-7 (2008).

6. Orru, R., Pazmiño, D.E., Fraaije, M.W. & Mattevi, A. Joint functions of protein residues and NADP(H) in oxygen activation by flavin-containing monooxygenase. *J Biol Chem* **285**, 35021-8 (2010).

7. Cho, H.J. et al. Structural and functional analysis of bacterial flavin-containing monooxygenase reveals its ping-pong-type reaction mechanism. *Journal of Structural Biology* **175**, 39-48 (2011).

8. Kubitza, C. et al. Crystal structure of pyrrolizidine alkaloid N-oxygenase from the grasshopper Zonocerus variegatus. *Acta Crystallogr D Struct Biol* **74**, 422-432 (2018).

9. Naumann, C., Hartmann, T. & Ober, D. Evolutionary recruitment of a flavin-dependent monooxygenase for the detoxification of host plant-acquired pyrrolizidine alkaloids in the alkaloid-defended arctiid moth Tyria jacobaeae. *Proceedings of the National Academy of Sciences* **99**, 6085-6090 (2002).

10. Hansen, B.G., Kliebenstein, D.J. & Halkier, B.A. Identification of a flavin-monooxygenase as the S-oxygenating enzyme in aliphatic glucosinolate biosynthesis in Arabidopsis. *The Plant Journal* **50**, 902-910 (2007).

11. Suh, J.-K. & Robertus, J.D. Yeast flavin-containing monooxygenase is induced by the unfolded protein response. *Proceedings of the National Academy of Sciences* **97**, 121-126 (2000).

12. Zhang, M. & Robertus, J.D. Molecular cloning and characterization of a full-length flavin-dependent monooxygenase from yeast. *Archives of Biochemistry and Biophysics* **403**, 277-283 (2002).

13. Eswaramoorthy, S., Bonanno, J.B., Burley, S.K. & Swaminathan, S. Mechanism of action of a flavin-containing monooxygenase. *Proceedings of the National Academy of Sciences* **103**, 9832-9837 (2006).

14. Valentino, H. et al. Structure and function of a flavin-dependent S-monooxygenase from garlic (Allium sativum). *J Biol Chem* **295**, 11042-11055 (2020).

15. Yoshimoto, N. et al. Identification of a flavin-containing S-oxygenating monooxygenase involved in alliin biosynthesis in garlic. **83**, 941-951 (2015).

16. Riebel, A., de Gonzalo, G. & Fraaije, M.W. Expanding the biocatalytic toolbox of flavoprotein monooxygenases from Rhodococcus jostii RHA1. *Journal of Molecular Catalysis B: Enzymatic* **88**, 20-25 (2013).

17. Riebel, A., Fink, M.J., Mihovilovic, M.D. & Fraaije, M.W. Type II Flavin-Containing Monooxygenases: A New Class of Biocatalysts that Harbors Baeyer–Villiger Monooxygenases with a Relaxed Coenzyme Specificity. *ChemCatChem* **6**, 1112-1117 (2014).

18. Szolkowy, C., Eltis, L.D., Bruce, N.C. & Grogan, G. Insights into Sequence–Activity Relationships amongst Baeyer–Villiger Monooxygenases as Revealed by the Intragenomic Complement of Enzymes from Rhodococcus jostii RHA1. **10**, 1208-1217 (2009).

19. Yi, J., Liu, L., Cao, Y., Li, J. & Mei, M. Cloning, characterization and expression of OsFMO(t) in rice encoding a flavin monooxygenase. *Journal of Genetics* **92**, 471-480 (2013).

20. Zhao, Y. et al. A role for flavin monooxygenase-like enzymes in auxin biosynthesis. *Science* **291**, 306-9 (2001).

21. Hartmann, M. et al. Flavin Monooxygenase-Generated N-Hydroxypipecolic Acid Is a Critical Element of Plant Systemic Immunity. *Cell* **173**, 456-469.e16 (2018).

22. Thodberg, S. et al. A flavin-dependent monooxygenase catalyzes the initial step in cyanogenic glycoside synthesis in ferns. *Communications Biology* **3**, 507 (2020).

23. Agosin, M. & Ankley, G.T. Conversion of N,N-dimethylaniline to N,N-dimethylaniline-N-oxide by a cytosolic flavin-containing enzyme from Trypanosoma cruzi. *Drug Metabolism and Disposition* **15**, 200 (1987).

24. Romero, E., Castellanos, J.R.G., Mattevi, A. & Fraaije, M.W. Characterization and Crystal Structure of a Robust Cyclohexanone Monooxygenase. 55, 15852-15855 (2016).

25. Campbell, A.C. et al. Trapping conformational states of a flavin-dependent N-monooxygenase in crystallo reveals protein and flavin dynamics. J Biol Chem 295, 13239-13249 (2020).
